# Socioeconomic inequalities in waiting times for breast cancer surgery

**DOI:** 10.1002/hec.4906

**Published:** 2024-10-03

**Authors:** Maria Ana Matias, Rita Santos, Luigi Siciliani, Peter Sivey, Andrew Proctor

**Affiliations:** ^1^ Centre for Health Economics University of York York UK; ^2^ Department of Economics and Related Studies University of York York UK; ^3^ Department of Oncology York and Scarborough Teaching Hospitals NHS Foundation Trust York UK

**Keywords:** access, breast cancer, inequalities, waiting times

## Abstract

Prompt access to cancer care is a policy priority in several OECD countries, because delayed access can exacerbate deleterious health outcomes. Access to care based on need remains a key pillar of publicly‐funded health systems. This study tests for the presence of inequalities in waiting times by socioeconomic status for patients receiving breast cancer surgery (mastectomy or breast conserving surgery) in England using the Hospital Episode Statistics. We investigate separately the pre‐COVID‐19 period (April 2015–January 2020), and the COVID‐19 period (February 2020–March 2022). We use linear regression models to study the association between waiting times and income deprivation measured at the patient's area of residence. We control for demographic factors, type and number of comorbidities, past emergency admissions and Healthcare Resource Groups, and supply‐level factors through hospital fixed effects. In the pre‐COVID‐19 period, we do not find statistically significant associations between income deprivation in the patient's area of residence and waiting times for surgery. In the COVID‐19 period, we find that patients living in the most deprived areas have longer waiting times by 0.7 days (given a mean waiting time of 20.6 days).

## 1 INTRODUCTION

In many publicly‐funded health systems, prompt access to care is a policy priority and patients on the list are prioritised based on need and not by ability to pay or socioeconomic status (OECD, 2020). Access to cancer care in particular is a policy priority in several OECD countries, because long waiting times can exacerbate deleterious health outcomes, including higher mortality, which can contribute to health inequalities. Several countries have introduced shorter cancer‐specific maximum waiting time targets to ensure cancer patients are prioritised on the waiting list (OECD, 2020). For a given level of need, patients with different socioeconomic status can vary in how long they wait, which is a dimension of patient responsiveness.

In this study, we test for the presence of inequalities in waiting times by socioeconomic status for women who had been diagnosed with breast cancer in England and required either a mastectomy or breast conserving surgery. We focus on breast cancer because this is one of the most common cancers and the most common cause of death by cancer for women in the UK (Cancer Research UK, 2023). We test for wait inequalities in two separate periods: the pre‐COVID‐19 period, between April 2015 and January 2020, and the COVID‐19 period, between February 2020 and March 2022. At the beginning of the pandemic in 2020 there were disruptions of screening and urgent cancer referrals (Duffy et al., 2022; Watt et al., 2022) and these disruptions could differ amongst patients by socioeconomic status. With screening temporarily suspended, patients could only be diagnosed on a symptomatic pathway, which reduces the total number of diagnoses in the short run until screening resumes. Even patients with a breast symptom may not present if they were worried to come to a health care setting where the virus was concentrated, they were worried to leave the house due to government advice, or believed that cancer services were not available, which can differ by socioeconomic status. Less screening for patients with lower socioeconomic status could lead to delays in severe cancers being diagnosed. We might therefore expect waiting time inequalities to differ during the pandemic.

We measure socioeconomic status through an index of income deprivation attributed to patients at the small geographical area, known as the Lower Super Output Area (LSOA) level. We control for a rich set of patient characteristics that may confound the relationship between waiting times and socioeconomic status. These include age, gender, ethnicity, type of breast cancer (whether they have early stage of breast cancer, i.e. Ductal carcinoma in situ [DCIS], or invasive breast cancer), number and type of secondary diagnoses (including personal or family history of malignant neoplasm), and recent history of emergency care utilisation.

We control for systematic differences in waiting times across hospitals by including hospital fixed effects in line with previous literature (e.g., Laudicella et al., 2012; Moscelli et al., 2016). Differences in waiting times across hospitals may be due to demand factors (e.g., a higher proportion of an elderly population) and supply factors (hospital differences in quality, capacity and efficiency) (Brindley et al., 2023). Therefore, our estimates can be interpreted as inequalities in waiting times across patients who attend the same hospital but live in areas with different deprivation.

We also test whether the waiting time inequalities by income deprivation differ across two main diagnoses, and replicate the analysis for two sub‐samples: (i) patients with DCIS, and (ii) patients with invasive breast cancer. Similarly, we test whether waiting time inequalities differ across two main procedures, and replicate the analysis for two sub‐samples: (i) patients receiving breast conserving surgery, and (ii) patients receiving a mastectomy. Given that more deprived patients tend to be in worse health, we investigate the role played by comorbidities and past emergency admissions in explaining the association between waiting times and income deprivation.

In the pre‐COVID‐19 period (April 2015–January 2020), we find that mean (unadjusted) waiting time across income deprivation quintiles is very similar, ranging from 19.9 days for the most deprived quintile to 20.1 days for the least deprived quintile. However, because more deprived patients tend to be in worse health, the lack of differences in waiting times do not necessarily imply there are no socioeconomic inequalities once controlling for patient characteristics. Moreover, hospitals can differ in waiting times due to demand or supply factors.

Our main regression results show that after controlling for a range of patient characteristics (including comorbidities and past emergency admissions) and systematic differences across hospitals (by including hospital fixed effects), patients living in areas with higher income deprivation are not statistically significantly associated with inpatient waiting times for breast cancer surgery in the pre‐COVID‐19 period. However, we do find that patients with past emergency admissions, who have a more complex clinical background, tend to wait longer and that more deprived patients have a higher prevalence of past emergency admissions and comorbidities.

The results are qualitatively similar in the pre‐COVID‐19 period when we split the sample by the two diagnoses (patients with DCIS, and patients with invasive breast cancer) or we split the sample by the two procedures (patients receiving breast conserving surgery and patients receiving a mastectomy).

We extend our models to the period after the onset of the COVID‐19 pandemic (February 2020–March 2022). After controlling for patient characteristics and systematic differences across hospitals, we find that living in the most income‐deprived areas is associated with a longer waiting time by 0.7 days. The results are similar for patients with invasive cancer, who account for 89% of the sample, and those receiving either breast conserving surgery or mastectomy.

We also compare results when we exclude hospital fixed effects from our regression model to infer the extent to which systematic differences in waiting times across hospitals contribute to inequalities in waiting times by income deprivation. For the COVID‐19 period, income deprivation is not associated with waiting times when excluding hospital fixed effects. This finding is in contrast with the model with hospital fixed effects, which suggests that more deprived patients wait up to 0.7 days longer. One explanation for these contrasting findings is that more deprived patients are treated on average by hospitals that have systematically shorter waiting times but this effect is offset by the waiting time inequalities arising within a hospital. In the pre‐COVID‐19 period, there is no association between waiting times and deprivation both with and without hospital fixed effects.

These findings highlight the need for targeted interventions to mitigate inequalities that emerged during COVID‐19, such as policies that reduce barriers to timely surgery for patients living in more deprived areas. Additionally, better coordination is crucial for patients with complex health histories. Developing an integrated care pathway could smooth pre‐surgical processes and minimise delays caused by additional appointments and checks.

We contribute to the existing literature in several ways. First, we test for waiting time inequalities for cancer patients, where the literature is more limited (see related literature section). Waiting times for elective cancer surgery are relatively short in comparison to less urgent elective operations such as joint replacements or cataract surgery. However, any differences in waiting times are potentially consequential due to the risk of cancer worsening over time. Second, we test for waiting time inequalities before and after the onset of the COVID‐19 pandemic. As the pandemic disrupted cancer care, there is the potential for lengthened waiting times and worse inequalities after the onset of the pandemic.

## 2 RELATED LITERATURE

There is a growing literature documenting the presence of inequalities in waiting times by socioeconomic status for non‐emergency treatments (Landi et al., 2018; Siciliani, 2016). In the English National Health Service (NHS), in which care is free at the point of use and largely funded by general taxation, there is evidence that patients with higher income deprivation wait longer for common procedures, such as hip replacement (Laudicella et al., 2012) and coronary bypass (Moscelli et al., 2018). This evidence suggests that these inequalities arise within hospitals as opposed to across hospitals. There is also evidence of wait inequalities for publicly‐funded patients in other countries, such as for Norway in relation to hip replacement (Monstad et al., 2014) and all elective care (Kaarboe & Carlsen, 2014), and for Australia (Johar et al., 2013; Sharma et al., 2013). In contrast, one study for Denmark did not find wait inequalities for several common elective surgical procedures (Simonsen et al., 2020).

Less is known in relation to waiting time inequalities for more urgent conditions, such as cancer care. Saito et al. (2021) find no differences in waiting time for colon cancer surgery by local area income deprivation. Bosque‐Mercader et al. (2023) compare waiting time inequalities in Spain between six common planned surgeries (hip and knee replacement, cataract surgery, hysterectomy, prostatectomy, and coronary bypass) and four cancer surgeries (prostate, breast, colorectal, and lung cancer surgery). They provide some evidence of socioeconomic inequalities for hip replacement, cataract surgery, hysterectomy, and breast cancer surgery, though the quantitative effect is relatively small. They find no differences for the other procedures.

A systematic review suggests that longer waiting times for cancer treatment are associated with higher mortality rates (Hanna et al., 2020). There is evidence in England that shorter waiting times can lead to better diagnosis and less costly treatment (Sun et al., 2020). Conversely, the predicted impact of delays in time‐to‐treatment initiation results in excess mortality (Figueroa et al., 2021). However, one study in England did not find an association between waiting times and 5‐year survival within the overall target of 62 days from referral to treatment (Redaniel et al., 2013). In Italy, the postponement of screening during COVID‐19 was associated with an increase in nodal involvement and stage three cancers at diagnosis (Toss et al., 2021).

There is a lack of studies specifically looking at the variation in waiting time for breast or other cancer patients, and how it relates to socioeconomic status. Most of the literature is concerned with exploring broader social or demographic variations in access to care and treatment (Corner & Brindle, 2011; Gu et al., 2018). Gu et al. (2018) reviews the evidence on the determinants of treatment by breast‐conserving surgery versus mastectomy for early‐stage breast cancer, finding that several studies indicate an association between low socioeconomic status and higher mastectomy rates. Both Downing et al. (2007) and Raine et al. (2010) use English data and show that treatment by mastectomy as opposed to breast‐conserving surgery is associated with socioeconomic deprivation at the small‐area level. Downing et al. (2007) also show an association between low socioeconomic status and late‐stage diagnosis and Raine et al. (2010) also shows that patients from deprived areas are much more likely to be admitted as an emergency for breast cancer treatment. Aarts et al. (2012) produce some similar findings for the Netherlands. Similar findings are also available for other types of cancer, for example, in rates of resection for lung cancer (Forest et al., 2013). One study in Canada shows across a range of different common cancers that lower socioeconomic status is associated with diagnosis at a later stage (Booth et al., 2009) and a study from Australia shows breast cancer survival rates are higher for publicly‐funded patients living in more socio‐economically advantaged areas (Dasgupta et al., 2012).

This paper contributes to the existing literature by presenting a detailed analysis of inequalities in waiting times for patients undergoing breast cancer surgery. Additionally, it investigates the potential impact of the COVID‐19 pandemic on these disparities by comparing waiting times before and after the onset of the pandemic. Given the significant disruptions to cancer care caused by the pandemic, our analysis explores the extent to which waiting times may have lengthened and inequalities may have worsened during this period.

## 3 INSTITUTIONAL BACKGROUND

The English NHS provides universal access to healthcare, which is free at the point of use. To access specialist care, such as cancer treatment, patients need a referral from their general practitioner (GP), which may lead to an outpatient appointment and subsequent surgery if deemed appropriate. Patients typically experience a waiting time before the initial specialist appointment (outpatient waiting time) and any subsequent hospital treatment (inpatient waiting time). Waiting times for care are the focus of targets, maximum waiting time guarantees and other policies in the NHS aimed at reducing waiting times, similarly to other OECD countries (OECD, 2020).

Figure A1 in the Appendix, provides the typical care pathway for breast cancer patients (NHS England, 2016a). The most common pathway is via an urgent GP referral to the hospital specialist, followed by a first specialist appointment, which may lead to a diagnosis and a decision to treat. There will be a further wait until treatment or surgery. The second most common route is for patients to be referred to a specialist following diagnosis via a routine breast screening appointment. In 2018, 51% of new breast cancer diagnoses were an urgent referral from a GP, 33% were following routine breast screening and the remaining 16% were following non‐urgent GP referrals or other sources (e.g., referrals from the emergency department) (Cancer Research UK, 2023).

The route to diagnosis and treatment is associated with stage of diagnosis, with screening identifying more early‐stage cancers and urgent GP referral identifying more later‐stage cancers. Of breast cancer diagnosed via GP urgent referral 29% were at stage 1% and 64% at stage 2, and of those diagnosed via screening, 65% were at stage 1% and 26% at stage 2 (Cancer Research UK, 2023). By stage, the data show that of all stage 1 breast cancers, 51.8% are diagnosed via screening and 36.3% via urgent GP referral; and of all stage 2 breast cancers 22.7% are diagnosed via screening and 65.3% via urgent GP referral (National Cancer Registration and Analysis Service, 2024).

National Institute for Health and Care Excellence, 2021 provides guidance about which patients should be put on an urgent cancer pathway by age and genetic history of the patient. For example, patients aged under 30 with an unexplained breast lump are only referred for a non‐urgent appointment with a specialist.

Breast screening in the UK is recommended every three years (Cancer Research UK, 2023) for women aged over 50 and has been shown to be highly effective at reducing breast cancer deaths (Marmot et al., 2013). All women over 50 are invited for routine screening appointments every three years where a mammogram is taken of each breast. Results should be received within two weeks and follow up tests (biopsy or ultrasound) should have results received within a further week (NHS, 2021b). When a cancer diagnosis is confirmed, patients will be referred for treatment, for example, an outpatient appointment where the patient may be put on the waiting list for surgery.

In 2000 the NHS Cancer Plan set a maximum waiting time guarantee of *two weeks* for the initial wait from GP urgent referral to a specialist appointment, *one month* from cancer diagnosis to treatment (the inpatient waiting time), and *two months* for the overall wait from urgent GP referral to treatment (NHS Executive, 2000). One additional target has been in place since 2010, which relates explicitly to breast cancer. It specifies a 14‐day target from GP referral to first specialist appointment for symptomatic patients who were not initially suspected of breast cancer (NHS Improvement, 2010).

Cancer waiting times have been routinely reported by cancer type and NHS Hospital Trust.^1^ The indicators include the proportion of patients waiting less than two months (62 days) from referral to treatment, less than one month (31 days) from diagnosis to treatment, and less than 14 days for symptomatic breast patients not initially suspected of cancer. In 2019/20, 89.4% of breast cancer patients waited less than 62 days for treatment following an urgent referral from a GP, and 97% of patients waited less than 31 days between the decision to treat and treatment (the inpatient waiting time). In this study we use data on inpatient wait, the time between the decision to treat and treatment, as the measure of waiting time.

During the COVID‐19 pandemic, a series of supply‐side and demand‐side responses affected the provision of cancer care. On the supply‐side, on the March 17, 2020 NHS England and Improvement issued a letter to NHS providers and Clinical Commissioning Groups (CCGs)^2^ bodies requiring hospitals to free up maximum inpatient and critical care capacity (Stevens & Pritchard, 2020). The letter led to the postponement of non‐urgent elective care operations and a move to providing remote consultations for outpatient appointments and primary care, while emergency admissions, cancer treatment and other clinically urgent care should have continued as normal. This was followed by a period of encouraging the NHS to return to higher levels of activity in the summer of 2020 (Stevens & Pritchard, 2020). However, in the winter of 2020/21 there was a second wave of COVID‐19 infections in the UK and the NHS returned to the highest level of emergency preparedness (Willett, 2020), which led to a decline in non‐COVID activities.

On the demand‐side, there were large falls in GP consultations and hospital A&E attendances throughout the months of 2020 and 2021 most affected by the pandemic (Burn et al., 2021; Fraser & Fisher, 2021). New urgent referrals for breast cancer were 7% lower than expected from April 2020 to the end of January 2021, and in the same period, first treatments for breast cancer were 25% lower than expected levels (Watt et al., 2022). The NHS was slow to recover from this disruption, with the proportion of patients waiting more than 104 days for cancer treatment following an urgent referral at record levels (10.7%) in early 2022 (Clover, 2022).

Routine data collected by the NHS shows some differences in diagnosis and outcomes by socioeconomic status attributed from small areas. Pre‐COVID‐19 data show a much smaller number of breast cancer diagnoses (6016 in 2019), higher proportion diagnosed at stage 3 or 4 (16.0% in 2019) and lower 5‐year survival (82.7%) in the most‐deprived areas of England compared to the least deprived areas (9186 diagnoses, 12.9% at stages 3 or 4 and 88.2% 5‐year survival) (National Cancer Registration and Analysis Service, 2024). Research has also shown a negative correlation between area‐level deprivation and breast screening rates (Bhola et al., 2015; Kurani et al., 2020; Mottram et al., 2021; Smith et al., 2019).

## 4 DATA

We use data from Hospital Episode Statistics (HES), an administrative database covering patients admitted to hospital in England between 2015/16 and 2021/22. Our unit of analysis is the inpatient spell defined as a continuous patient stay in one of the NHS Hospital Trusts.^3^ Our sample includes all female patients whose primary diagnosis was breast cancer, either invasive breast cancer (International Classification of Diseases 10^th^ Revision [ICD‐10] code C50, “malignant neoplasm of breast”) or DCIS of the breast (DCIS) (ICD‐10 code D05, “carcinoma in situ of breast”), and underwent surgery, either mastectomy (Operating Procedure Codes Supplement version 4.5 [OPCS‐4.5] code B27) or breast conserving surgery (OPCS‐4.5 code B28) and which fall within Healthcare Resource Group (HRG) subchapter JA “Breast Disorders and Procedures”. We exclude patients who had a previous hospital admission for breast cancer in the previous two years.^4^

Our outcome is the *inpatient waiting time*, which is defined as the difference between the time the patient is added to the waiting list (decision to treat in Figure A1), after specialist assessment, and the time the patient is admitted to the hospital for surgery. Waiting time is measured in days.

In terms of patients' characteristics, HES has information on patient's age at admission, which we measure in 5‐year bands, ethnicity, primary diagnosis (invasive breast cancer vs. Ductal carcinoma in situ, classified in the ICD‐10 as C50 and D05, respectively), secondary diagnoses (dummy variables for the most frequent individual secondary diagnoses, comprising each more than 1% of patients, and for the number of secondary diagnoses), main surgical procedure (mastectomy or breast‐conserving surgery, coded in OPCS‐4.5 as B27 and B28, respectively) and HRG (using the 5‐digit code, within subchapter JA “Breast Disorders and Procedures”). We also measure the number of patient's past emergency admissions in the year preceding the breast cancer elective admission. The secondary diagnoses were used as a proxy for comorbidities.

Socioeconomic status (SES) is proxied by the income deprivation quintile measured at the patient's LSOA^5^ of residence available from the GOV.UK (2019), which is based on a broad set of indicators related to income.

We perform the analysis separately for the pre‐COVID‐19 period (April 2015–January 2020) and for the COVID‐19 period (February 2020–March 2022). Our pre‐COVID‐19 sample includes 182,356 female patients treated in 140 NHS Hospital Trusts. We drop 1124 patients without a record of their LSOA. We also dropped 1867 patients with waiting time above 72 days and 1843 patients with a length of stay (LOS) above 8 days,^6^ equivalent to 2.6% of the initial sample.

The COVID‐19 sample includes 72,816 female patients treated in 131 NHS Trusts. We drop 592 patients without a record of their LSOA. We drop outliers in terms of waiting times (1380 patients with waiting time above 72 days) and LOS (477 patients with a LOS above 7 days),^7^ equivalent to 2.5% of the initial sample.

## 5 METHODS

We use the following regression model to analyze socioeconomic inequalities in inpatient waiting times for breast cancer:

(1) $$w_{ijt}={\text{SE}S^{\prime }}_{i}\;\beta +x_{i}^{\prime }\;\gamma +d_{j}+d_{t}+d_{m}+\varepsilon_{ijt}$$

where *w*_*ijt*_ is waiting time (measured in days) for patient *i*, in hospital *j*, in year *t*. *SES*_*i*_ is a vector of variables related to patient socioeconomic status, measured as quintiles of income deprivation (with a dummy variable for each quintile, and using the least deprived quintile as the reference group).

*x*_*i*_ is a vector of patient characteristics to control for patient casemix because some of the gradient may be due, for example, to patients with lower SES being in worse health being prioritised on the waiting list. These include age, ethnicity, primary diagnosis, secondary diagnoses, number of comorbidities, past emergency admissions, and type of procedure.

$d_{j}$, $d_{t}$ and $d_{m}$ are the hospital, year and month fixed effects, respectively. Hospital fixed effects ($d_{j}$) control for hospital (demand and supply) factors. The gradient can therefore be interpreted as inequalities arising within rather than across hospitals. However, to understand the role of supply factors in explaining inequalities we also provide regression results where we exclude hospital fixed effects. The time fixed effects $d_{t}$ control for the time trend (e.g., due to technology development) and the month fixed effects $d_{m}$ control for seasonality. $\varepsilon_{ijt}$ is the error term. We estimate (1) by Ordinary Least Squares. We cluster robust standard errors at the hospital level.

## 6 RESULTS

### 6.1 Descriptive statistics

Table 1 provides descriptive statistics for patients who were diagnosed with breast cancer and received a mastectomy or breast conserving surgery. In the pre‐COVID‐19 period (April 2015–January 2020), the average inpatient waiting time in the sample period is 20.1 days, which increased to 20.6 days in the COVID‐19 period (February 2020–March 2022).

**TABLE 1:** Descriptive statistics.

	Pre‐COVID‐19		COVID‐19	
	*N* = 182,356		*N* = 72,816	
	Mean	Std. dev.	Mean	Std. dev.
Inpatient waiting time (days)	20.06	11.568	20.60	12.184
Type of procedure				
Mastectomy (B27)	0.316	0.465	0.315	0.464
Breast conserving surgery (B28)	0.684	0.465	0.685	0.464
Primary diagnosis				
Invasive breast cancer (C50)	0.880	0.324	0.885	0.319
Ductal carcinoma in situ of breast (D05)	0.120	0.324	0.115	0.319
Income deprivation score (quintiles)				
Least income deprived quintile	0.237	0.425	0.240	0.427
2nd Income deprived quintile	0.225	0.417	0.230	0.421
3rd Income deprived quintile	0.211	0.408	0.210	0.407
4th Income deprived quintile	0.18	0.384	0.178	0.383
Most income deprived quintile	0.148	0.355	0.143	0.350
Age (years)	61.36	12.71	61.51	12.81
[16,44]	0.089	0.285	0.096	0.295
[45,49]	0.096	0.295	0.078	0.268
[50,54]	0.135	0.342	0.132	0.338
[55,59]	0.124	0.33	0.136	0.342
[60,64]	0.128	0.334	0.135	0.342
[65,69]	0.151	0.358	0.140	0.347
[70,74]	0.118	0.322	0.115	0.319
[75,79]	0.078	0.268	0.086	0.281
[80,102]	0.081	0.272	0.082	0.275
Ethnicity				
White	0.8	0.4	0.746	0.435
Mixed	0.005	0.072	0.007	0.081
Asian	0.033	0.178	0.035	0.184
Black	0.017	0.129	0.018	0.132
Others	0.017	0.127	0.020	0.141
Missing	0.129	0.335	0.174	0.379
Secondary diagnosis/Comorbidities				
C77 ‐ secondary and unspecified malignant neoplasm of lymph nodes	0.195	0.396	0.200	0.400
D05 ‐ ductal carcinoma in situ of breast	0.042	0.2	0.058	0.233
Z85 ‐ personal history of malignant neoplasm	0.064	0.245	0.077	0.266
Z80 ‐ family history of malignant neoplasm	0.072	0.258	0.092	0.290
Z86 ‐ personal history of certain other diseases	0.181	0.385	0.221	0.415
Z87 ‐ personal history of other diseases and conditions	0.033	0.18	0.054	0.226
Z88 ‐ personal history of allergy to drugs, medicaments and biological substances	0.109	0.311	0.135	0.342
Z90 ‐ acquired absence of organs, not elsewhere classified	0.067	0.25	0.095	0.294
Z92 ‐ personal history of medical treatment	0.128	0.334	0.158	0.365
Z96 ‐ presence of other functional implants	0.037	0.19	0.046	0.210
Z11‐ encounter for screening for infectious and parasitic diseases			0.031	0.174
E03 ‐ other hypothyroidism	0.074	0.262	0.079	0.270
E11 ‐ non‐insulin‐dependent diabetes mellitus	0.078	0.268	0.079	0.269
E66 ‐ obesity due to excess calories	0.118	0.322	0.166	0.372
E78 ‐ disorders of lipoprotein metabolism and other lipidaemis	0.052	0.222	0.063	0.243
I10 ‐ essential (primary) hypertension	0.283	0.451	0.278	0.448
I25 ‐ chronic ischemic heart disease	0.027	0.162	0.027	0.163
I48 ‐ atrial fibrillation and flutter	0.03	0.171	0.032	0.176
J44 ‐ other chronic obstructive pulmonary disease	0.032	0.177	0.033	0.179
J45 ‐ predominantly allergic asthma	0.098	0.297	0.103	0.304
K21 ‐ gastro‐oesophageal reflux disease	0.057	0.231	0.074	0.263
M13 ‐ other arthritis	0.036	0.185	0.031	0.173
M19 ‐ other arthrosis	0.041	0.199	0.054	0.227
F17 ‐ mental and behavioral disorders due to use of tobacco	0.09	0.286	0.080	0.271
F32 ‐ depressive episode	0.057	0.232	0.070	0.256
F41 ‐ other anxiety disorders	0.06	0.237	0.088	0.283
Z13 ‐ special screening examination for other diseases and disorders	0.035	0.183	0.030	0.169
Other diagnoses	0.476	0.499	0.561	0.496
Number of secondary diagnoses/Comorbidities				
None	0.147	0.354	0.115	0.319
One diagnosis	0.188	0.391	0.153	0.360
Two diagnoses	0.177	0.382	0.155	0.362
Three diagnoses	0.142	0.349	0.139	0.345
Four diagnoses	0.109	0.311	0.115	0.319
Five diagnoses	0.077	0.266	0.089	0.285
More than six diagnoses	0.16	0.367	0.234	0.424
Number of past emergency admissions (<365 days)				
None	0.887	0.316	0.884	0.320
One or two past emergency admissions	0.098	0.297	0.102	0.303
More than two past emergency admissions	0.015	0.121	0.014	0.117

*Note*: Pre‐COVID‐19 period: April 2015 to January 2020; COVID‐19 period: February 2020 to March 2022. Breast cancer patients who had surgery: 2015/16–2021/22.

In the pre‐COVID‐19 period, 88% of patients were diagnosed with invasive breast cancer (C50), while the remaining 12% were diagnosed with DCIS (carcinoma in situ, D05). 32% had a mastectomy and 68% had breast conserving surgery. Patients were on average 61 years old. 89% of patients had no past emergency admissions in the year preceding the surgery, 10% had one or two, and only 1.5% had more than two past emergency admissions. 15% of patients had no comorbidities, 37% had one or two, 33% had three to five, and 16% had more than six comorbidities. In terms of secondary concurrent diagnoses, 20% of patients were diagnosed with secondary and unspecified malignant neoplasm of lymph nodes (C77); 6% had a personal history of malignant neoplasm (Z85); 7% had family history of malignant neoplasm (Z80); 28% had hypertension (I10); 13% had a personal history of medical treatment (Z92), mostly involving chemotherapy. Patients from the most income‐deprived quintile received 15% of surgeries, and those in the least income‐deprived quintile instead received 24% of surgeries (with 22%, 21% and 18% respectively in the second, third and fourth income‐deprivation quintile). 80% of patients are White, 3.3% are Asian and 1.7% are Black.

In the COVID‐19 period, patients' characteristics are generally comparable to the pre‐COVID‐19 period in terms of primary diagnosis, secondary diagnoses, income deprivation, age, and past emergency admissions. The proportion of White patients is 74.6%, which is lower than in the pre‐COVID‐19 period (at 80%), while the proportion of patients without a recorded ethnicity is 17.4% (which is higher than in the pre‐COVID‐19 period at 12.9%). The proportion of patients with more than six secondary diagnoses is 23.4%, which is higher than in the pre‐COVID‐19 period (at 16%), while the proportion with no secondary diagnoses is 11.5%, which is lower than in the pre‐COVID‐19 period (at 14.7%).

**FIGURE 1:**
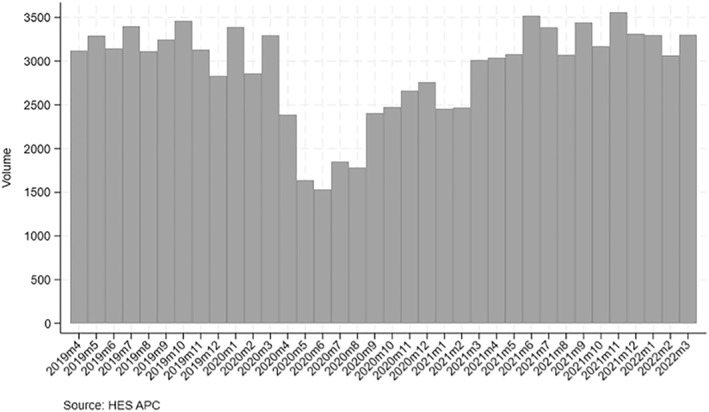
Monthly volume in 2019/20–2021/22.

**FIGURE 2:**
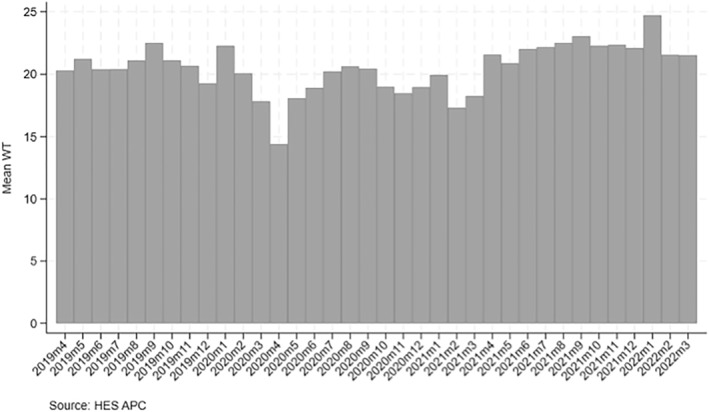
Monthly mean waiting time in 2019/20–2021/22.

Figures 1 and 2 depict the volume of surgery and mean waiting time by month from April 2019 to March 2022. Total volume between April 2020 and March 2021 was 27,423 surgeries, which is 29% lower relative to the total volume in the previous 12 months (equal to 38,279 surgeries) and with the largest drop in volume concentrated in May‐August 2020. Between April 2021 and March 2022, the number of surgeries returned to pre‐pandemic levels.

Overall, despite the reduction in surgeries during the COVID‐19 period, this decrease was evenly distributed across socioeconomic deprivation groups, as indicated by the consistent proportions across these groups. This is confirmed in Table 1, which shows that the proportion of patients who had breast cancer surgery by deprivation is very similar in the COVID‐19 period (e.g., 14.3% vs. 14.8% for the most deprived, and 24.0% vs. 23.7% for the least deprived).

The mean waiting time between April 2020 and March 2021 was 18.6 days, which is lower than in the previous year (20.6 days). Between April 2021 and March 2022, the mean waiting time increased by 3.6–22.2 days.

### 6.2 Regression results

Table 2 provides the regression results for our preferred specification, which includes year, month and hospital fixed effects, in addition to controlling for age, primary diagnosis, secondary diagnoses, number of comorbidities, past emergency admissions, type of procedure, and HRGs (omitted from the table).^8^

**TABLE 2:** Regression results.

	(1) Waiting times Pre‐COVID‐19	(2) Waiting times COVID‐19
Income deprivation (baseline 1 ‐ least deprived)		
2nd Income deprived quintile	0.0703	0.137
3rd Income deprived quintile	0.0258	0.239*
4th Income deprived quintile	0.0855	0.430**
Most income deprived quintile	0.114	0.657***
Age groups (baseline <45)		
[45,49]	0.429**	−0.429*
[50,54]	0.640***	0.185
[55,59]	0.793***	0.328
[60,64]	0.980***	0.428*
[65,69]	0.981***	0.555*
[70,74]	0.988***	0.522*
[75,79]	0.986***	0.309
[80,102]	1.695***	1.582***
Ethnicity (baseline = White)		
Mixed	−0.125	−0.846
Asian	−0.463*	−0.152
Black	0.0742	−0.343
Others	−0.14	−0.145
Missing	−0.279*	−0.345*
Mastectomy (B27)	−0.0555	−0.0366
Invasive breast cancer (C50)	−0.767***	−0.781***
C77 ‐ secondary and unspecified malignant neoplasm of lymph nodes	−0.316**	−0.210
D05 ‐ ductal carcinoma in situ of breast	−0.493**	−0.309
Z85 ‐ personal history of malignant neoplasm	−0.506***	−0.511**
Z80 ‐ family history of malignant neoplasm	−0.221	−0.274
Z86 ‐ personal history of certain other diseases	0.000803	−0.128
Z87 ‐ personal history of other diseases and conditions	−0.298	−0.104
Z88 ‐ personal history of allergy to drugs, medicaments and biological substances	−0.0722	−0.0548
Z90 ‐ acquired absence of organs, not elsewhere classified	−0.178	−0.260
Z92 ‐ personal history of medical treatment	2.631***	2.310***
Z96 ‐ presence of other functional implants	−0.146	−0.0339
Z11‐ encounter for screening for infectious and parasitic diseases		0.286
E03 ‐ other hypothyroidism	−0.0436	−0.120
E11 ‐ non‐insulin‐dependent diabetes mellitus	0.0302	0.519**
E66 ‐ obesity due to excess calories	0.0225	0.173
E78 ‐ disorders of lipoprotein metabolism and other lipidaemis	−0.167	0.135
I10 ‐ essential (primary) hypertension	0.0027	0.0669
I25 ‐ chronic ischemic heart disease	0.348	0.382
I48 ‐ atrial fibrillation and flutter	−0.466*	−0.425
J44 ‐ other chronic obstructive pulmonary disease	0.559***	0.488*
J45 ‐ predominantly allergic asthma	−0.122	−0.0555
K21 ‐ gastro‐oesophageal reflux disease	−0.0764	−0.108
M13 ‐ other arthritis	−0.314	0.235
M19 ‐ other arthrosis	−0.0198	−0.129
F17 ‐ mental and behavioral disorders due to use of tobacco	−0.173	0.0701
F32 ‐ depressive episode	−0.00392	0.155
F41 ‐ other anxiety disorders	−0.325**	−0.233
Z13 ‐ special screening examination for other diseases and disorders	1.388	2.180**
Other diagnoses	−0.123	0.137
Secondary diagnoses/comorbidities (baseline = 0)		
One diagnosis	0.435***	−0.117
Two diagnoses	0.687***	0.278
Three diagnoses	0.885***	0.275
Four diagnoses	0.981***	0.435
Five diagnoses	0.904***	0.440
More than six diagnoses	1.035***	0.573
Past emergency admissions (baseline = 0)		
Between one and two	2.153***	1.545***
Above two	3.785***	1.474**
Financial year (baseline 2015/16)		
2016/17	0.0897	
2017/18	−0.224	
2018/19	0.631*	
2019/20	0.951**	
Financial year (baseline 2019/20)		
2020/21		0.317
2021/22		2.642***
Month (baseline Apr)		
Jan	1.579***	3.769***
Feb	−0.843***	1.839***
Mar	−0.563**	1.342**
May	−0.102	1.086**
Jun	0.399*	1.898***
Jul	−0.0523	2.192***
Aug	0.29	2.618***
Sep	1.354***	2.883***
Oct	0.252	1.706***
Nov	0.105	1.742***
Dec	−0.751***	1.763***
Hospital fixed effects	Yes	Yes
HRGs effects	Yes	Yes
Observations	182,356	72,816
Adjusted *R* ^2^	0.118	0.164

*Note*: Linear regression model with clustered robust standard errors at the hospital level. HRGs: Healthcare Resource Groups. Pre‐COVID‐19 period: April 2015 to January 2020. COVID‐19 period: February 2020 to March 2022. Inpatient waiting time (days) and income deprivation.

*p < 0.05, ** *p* < 0.01, *** *p* < 0.001.

In the pre‐COVID‐19 period, relative to patients living in the least deprived areas, there are no statistically significant differences in inpatient waiting times across deprivation quintiles. The deprivation gradient is monotonic across quintiles and quantitatively small. For example, patients living in areas with highest income deprivation wait 0.11 days longer relative to patients living in the least deprived areas, but this is not statistically significant.

In relation to covariates, older patients tend to wait longer but the difference in waiting time is one day between the reference group (less than 45 years old) and the second oldest group (75 to 79). Relative to patients with invasive breast cancer, patients with DCIS have a longer waiting time by 0.8 days. Relative to White patients, those who are Asian have a shorter waiting time by 0.5 days.

Patients with more secondary diagnoses generally wait longer, but by at most one day. Similarly, patients with one or two past emergency admissions have a longer waiting time by 2.1 days compared to patients without any past emergency admissions. Patients with a personal history of malignant neoplasm (around 6% of patients) wait less by about half day.

In the COVID‐19 period, we find a pro‐rich gradient in waiting times. Patients in the most income deprived quintile wait 0.7 days longer relative to the least deprived while patients in the third and fourth quintiles wait 0.2 and 0.4 days longer, respectively. There are no statistically significant differences in waiting times between patients in the least deprived quintile relative to those in the second quintile.

In terms of patients characteristics, the results are broadly in line with those provided for the pre‐COVID‐19 period, except for the number of comorbidities and past emergency admissions. Specifically, there are no statistically significant differences in waiting times between patients with no comorbidities relative to those with comorbidities. Patients with more than two past emergency admissions wait 1.4 days longer relative to patients with no past emergency admissions, which is lower than in the pre‐COVID‐19 period (equal to 3.8 days). Waiting times were 2.6 days longer in 2021/22 compared to 2019/20.

The econometric specification in Table 2 includes hospitals' fixed effects, and the results can be interpreted in terms of inequalities arising within the hospitals: patients with different socioeconomic status attending the same hospital have potentially different waiting times. Some inequalities may arise also across hospitals if for example, patients in more deprived areas live closer to hospitals with shorter waiting times.

Table 3 presents the results of the association between waiting times and income deprivation when *excluding* hospitals' fixed effects, while still controlling for patient characteristics. The estimates can therefore be interpreted as reflecting inequalities that arise both within and across hospitals. For the pre‐COVID‐19 period the results show that income deprivation is not associated with waiting times, which is consistent with the model with hospital fixed effects (Table 2, column 1). These results suggest that systematic differences in waiting times across hospitals do not play a role in the association between waiting times and income deprivation in the pre‐COVID‐19 period.

**TABLE 3:** Regression results without hospital fixed effects.

	(1) Waiting time Pre‐COVID‐19	(2) Waiting time COVID‐19
Income deprivation (baseline 1 ‐ least deprived)		
2nd Income deprived quintile	−0.00684	−0.0467
3rd Income deprived quintile	0.0112	0.0540
4th Income deprived quintile	−0.166	−0.0968
Most income deprived quintile	−0.302	0.0461
Hospital fixed effects	No	No
HRGs effects	Yes	Yes
Observations	182,356	72,816
Adjusted *R* ^2^	0.029	0.051

*Note*: Linear regression model with clustered robust standard errors at the hospital level. All models include financial year and month fixed effects. We also control for age, ethnicity, type of diagnosis, type of procedure, secondary diagnosis, and past emergency admissions. HRGs: Healthcare Resource Groups. Pre‐COVID‐19 period: April 2015 to January 2020. COVID‐19 period: February 2020 to March 2022.

*p < 0.05, ** *p* < 0.01, *** *p* < 0.001.

For the COVID‐19 period, the results presented in Table 3 (column 2) show that income deprivation is not associated with waiting times. This finding is in contrast with the model with hospital fixed effects (Table 2, column 2) which suggests that patients in the most deprived quintile wait up to 0.7 days longer. The explanation for these contrasting findings is that patients living in areas with higher income deprivation are treated by hospitals that have systematically shorter waiting time. This difference in waiting times between hospitals is offset by the inequalities in waiting times within hospitals, where patients living in more deprived areas experience longer waits. Therefore, when hospital fixed effects are excluded, the income deprivation coefficients become statistically insignificant in Table 3.

These inequalities between hospitals may arise from factors such as variations in hospital resources, geographic location, or the demographics of the populations served. Conversely, inequalities within hospitals are likely influenced by internal administrative practices or prioritisation policies that may disproportionately impact certain patient groups, such as those from more socio‐economically deprived areas.

## 7 HETEROGENEITY ANALYSIS

In this section, we provide analysis with additional specifications and for different sub‐samples. We start by presenting the results for three additional regression models. In model (1) we control only for year and month fixed effects. This allows us to test whether mean waiting times for the least income deprived patients, which we observe in the descriptive statistics, are statistically significantly different from the other groups. In model (2), we introduce all covariates, including hospital/HRG fixed effects and some patient covariates, but exclude secondary diagnoses and past emergency visits. In model (3), we include all covariates as in the main model. The comparison of models (2) and (3) allows us to explore the role of secondary diagnoses and past emergency admissions in the waiting time gradient. We expect more deprived patients to be in poorer health, and therefore have more past emergency admissions and a higher incidence of diagnoses that can themselves be associated with longer or shorter waiting times and therefore contribute to the waiting time gradient.

Second, we conduct separate analyses for different diagnoses depending on whether the patient has been diagnosed with DCIS, which is considered the early stage of breast cancer, or with invasive breast cancer. This allows us to explore whether any income deprivation gradient differs by diagnosis.

Third, we conduct separate analyses by type of procedure depending on whether the patient has breast conserving surgery or, the more invasive, mastectomy.

Fourth, since distance to hospital could potentially affect how long patients wait, we run a robustness check where we add as an additional covariate the distance from where the patient resides to the hospital where the patient receives treatment.

All analyses are performed separately for the pre‐ and COVID‐19 periods.

### 7.1 Descriptive statistics by income deprivation

In the pre‐COVID‐19 period, waiting times are very similar across groups ranging from 19.9 days for the most deprived quintile to 20.1 days for the least deprived quintile (see Table A3 in the Appendix). A higher proportion of patients living in areas with higher income deprivation receive a mastectomy (34.3% vs. 30.1%). Patients living in more deprived areas have a higher prevalence of given comorbidities. For example, a higher proportion of patients living in more deprived areas have hypertension (32% vs. 26%), chronic heart disease (3.8% vs. 2%), had a depressive episode (8.4% vs. 4.2%) or suffer from anxiety (8.4% vs. 4.2%). Only 10.2% of patients living in more deprived areas had no secondary diagnoses in the year prior to admission, while this was 17.6% for patients living in least deprived areas. Similarly, 22.1% of patients living in areas with higher income deprivation had more than six diagnoses, while this was 12.8% for patients living in least deprived areas. Also, 14.4% of patients in the most deprived quintile had at least one emergency admission, while this was 9.5% for least deprived quintile.

In Table A4 in the Appendix, we present the same figures for the COVID‐19 period. Briefly, waiting times are similar across groups ranging from 20.7 days for the most deprived quintile to 20.6 for the least deprived quintile. Similar to the pre‐COVID‐19 period, patients living in more deprived areas receive a mastectomy (34.2% vs. 29.8%), have higher prevalence of comorbidities and past emergency admissions compared to patients living in the least deprived areas. Overall, for both periods, these figures suggest that patients living in areas with higher income deprivation are in poorer health.

### 7.2 Sequential inclusion of patient characteristics

The regression results for inpatient waiting times when we introduce the covariates sequentially can be found in Table A5 in the Appendix. For the pre‐COVID‐19 period, column (1) suggests that raw differences in waiting times between patients living in the least deprived areas and other deprivation groups are not statistically significant. Column (2) controls for hospital and HRG fixed effects but excludes past emergency admissions and secondary diagnoses. It suggests that patients living in the most‐income deprived areas wait 0.3 days more than patients living in the least income deprived areas. However, column (3) suggests that when controlling for those additional patient covariates (as in the main model) there are no statistically significant differences in waiting times across deprivation quintiles. These findings are consistent with patients living in more deprived areas having more past emergency admissions and a higher prevalence of secondary diagnoses, as shown in Table A3. Moreover, we know from Table 2 that a larger number of past emergency admissions is associated with a longer waiting time by about 2–4 days. Therefore, differences in waiting times by deprivation disappear once controlling for past emergency admissions.

For the COVID‐19 period, again column (1) suggests that raw differences in waiting times between patients in the least deprived areas and other deprivation groups are not statistically significant. Column (2) suggests that patients in the most income deprived areas wait 0.9 days more than patients in the least deprived areas, when we do not control for emergency admissions and secondary diagnosis, which reduces to 0.7 days when adding those covariates in column (3). Again, this is consistent with patients in more deprived areas having more past emergency admissions which are associated with longer waiting times.

### 7.3 DCIS versus invasive breast cancer

The descriptive statistics for waiting times by income deprivation quintiles when the sample is split by the two primary diagnoses, DCIS and invasive breast cancer both pre‐ and COVID‐19 periods can be found in Table A6 in the Appendix. For DCIS, the earliest form of breast cancer, which accounts for 12% of the sample, patients in the most deprived quintile wait less than patients in the least deprived quintile both pre‐COVID‐19 (19.8 vs. 20.5 days) and COVID‐19 (20.5 vs. 21.3 days). Differences in waiting times are smaller for the remaining part of the sample whose primary diagnosis is invasive breast cancer pre‐COVID‐19 (19.9 vs. 20.1), but patients in most deprived areas wait longer in the COVID‐19 period (20.8 vs. 20.4 days).

Table 4 presents the regression results in the pre‐COVID‐19 period.^9^ For the DCIS, column (1) suggests that differences in waiting times by income deprivation described in Table A6 are not statistically significant when only controlling for hospital fixed effects. Neither are there statistically significant differences across deprivation quintiles in the partial model in column (2), which includes most patient characteristics, or in the full model in column (3), which includes all patients characteristics. Unsurprisingly, the results for invasive breast cancer, which accounts for 88% of the sample, are very similar to those in Tables 2 and A5.

**TABLE 4:** Pre‐COVID‐19: Regression results by diagnosis.

	Ductal carcinoma in situ of breast			Invasive breast cancer		
	(1) Waiting time	(2) Waiting time	(3) Waiting time	(1) Waiting time	(2) Waiting time	(3) Waiting time
Income deprivation (baseline 1 ‐ least deprived)						
2nd Income deprived quintile	0.264	0.374	0.355	−0.0369	0.0784	0.0351
3rd Income deprived quintile	−0.251	−0.206	−0.245	0.0604	0.141	0.0571
4th Income deprived quintile	−0.150	0.275	0.211	−0.115	0.218*	0.0691
Most income deprived quintile	−0.679	0.0576	−0.0963	−0.112	0.383***	0.148
Hospital fixed effects	No	Yes	Yes	No	Yes	Yes
HRGs effects	No	Yes	Yes	No	Yes	Yes
Observations	21,802	21,802	21,802	160,554	160,554	160,554
Adjusted *R* ^2^	0.008	0.102	0.105	0.006	0.107	0.121

*Note*: Linear regression model with clustered robust standard errors at the hospital level. All models include financial year and month fixed effects. HRGs: Healthcare Resource Groups. Pre‐COVID‐19 period: April 2015 to January 2020. Specification model (1) does not include any controls; specification model (2) excludes past emergency admissions and secondary diagnosis; the full specification model is presented in (3).

*p < 0.05, ** *p* < 0.01, *** *p* < 0.001.

Table 5 provides the results for the COVID‐19 period. For the DCIS, we did not find any statistically significant differences in waiting times across deprivation quintiles in any of the three models. Given that about 89% of the patients were diagnosed with invasive breast cancer, the results are again unsurprisingly similar to those in Table 2 and Table A5. However, the coefficient on the most deprived quintile is somewhat larger. Patients in the most deprived quintile wait 1 day longer than patients in the least income deprived quintile in column (2), where we do not control for emergency admissions and secondary diagnoses, and reduces to 0.73 days in the full model.

**TABLE 5:** COVID‐19: Regression results by diagnosis.

	Ductal carcinoma in situ of breast			Invasive breast cancer		
	(1) Waiting time	(2) Waiting time	(3) Waiting time	(1) Waiting time	(2) Waiting time	(3) Waiting time
Income deprivation (baseline 1 ‐ least deprived)						
2nd Income deprived quintile	−0.0926	−0.0830	−0.118	−0.0196	0.195	0.162
3rd Income deprived quintile	−0.215	0.136	0.0635	0.173	0.370**	0.269*
4th Income deprived quintile	−0.234	0.304	0.171	0.0124	0.637***	0.464**
Most income deprived quintile	−0.848	0.241	0.0132	0.349	0.987***	0.733***
Hospital fixed effects	No	Yes	Yes	No	Yes	Yes
HRGs effects	No	Yes	Yes	No	Yes	Yes
Observations	8346	8346	8346	64,470	64,470	64,470
Adjusted *R* ^2^	0.019	0.148	0.151	0.030	0.157	0.167

*Note*: Linear regression model with clustered robust standard errors at the hospital level. All models include financial year and month fixed effects. HRGs: Healthcare Resource Groups. COVID‐19 period: February 2020 to March 2022. Specification model (1) does not include any controls; specification model (2) excludes past emergency admissions and secondary diagnosis; the full specification model is presented in (3).

*p < 0.05, ** *p* < 0.01, *** *p* < 0.001.

### 7.4 Breast conserving surgery versus mastectomy

Table A9 in the Appendix provides the mean waiting times by income quintiles when the sample is split by the two main types of surgery, breast conserving surgery and mastectomy both pre‐ and during COVID. In the pre‐COVID‐19 period, differences in waiting times by income deprivation are small. For breast conserving surgery, patients in the most income deprived quintile wait 0.1–0.2 days less relative to other income deprivation quintiles. For mastectomy, the two most deprived groups wait 0.2–0.3 days less than other groups. In the COVID‐19 period, patients living in the most income deprived areas who underwent breast conserving surgery wait 0.2 days more than patients in the least income deprived areas. For mastectomy, there is no difference in waiting times between the least and most income deprived quintiles, though the other groups wait less (by at most 0.3 days). In both periods, mean waiting time for breast conserving surgery is lower than for mastectomy. In the pre‐COVID‐19 period the mean difference in waiting time between both procedures is 1.1 days (19.7 vs. 20.8 days), in the COVID‐19 period that difference reduces to 0.7 days (20.4 vs. 21.1 days).

Table 6 presents the regression results for the pre‐COVID‐19 period. For breast conserving surgery, column (1) suggests that differences in waiting times by income deprivation described in Table A9 are not statistically significant. This is also the case for the full model in column (3). It is only in column (2), where past emergency admissions and secondary diagnoses are excluded from the control variables, that patients in the most deprived quintile wait 0.3 longer than the least deprived. A similar pattern emerges for mastectomy. Column (1) suggests that differences in waiting times by income deprivation described in Table A9 are not statistically significant. In column (2) patients in the fifth and fourth most income deprived quintiles undergoing mastectomy wait 0.5 and 0.3 days longer, respectively, relative to the least deprived if we do not control for the number of emergency admissions and secondary diagnosis. There are no statistically significant differences in the full model in column (3). These results are again in line with those presented in Table A5. These findings are consistent with patients living in more deprived areas having more past emergency admissions and a higher prevalence of secondary diagnoses. We know from Table 2 that more past emergency admissions is associated with a longer waiting time by about 2–4 days. Therefore, differences in waiting times by deprivation disappear once controlling for past emergency admissions. The results are similar when we restrict the analysis by procedure to patients with invasive breast cancer (see Table A11).

**TABLE 6:** Pre‐COVID‐19: Regression results by type of procedure.

	Breast conserving surgery			Mastectomy		
	(1) Waiting time	(2) Waiting time	(3) Waiting time	(1) Waiting time	(2) Waiting time	(3) Waiting time
Income deprivation (baseline 1 ‐ least deprived)						
2nd Income deprived quintile	0.0134	0.123	0.0873	−0.0752	0.0952	0.0381
3rd Income deprived quintile	0.00140	0.0564	−0.0122	0.0214	0.161	0.0717
4th Income deprived quintile	−0.102	0.185	0.0620	−0.247	0.314*	0.144
Most income deprived quintile	−0.214	0.270*	0.0597	−0.255	0.479**	0.232
Hospital fixed effects	No	Yes	Yes	No	Yes	Yes
HRGs effects	No	Yes	Yes	No	Yes	Yes
Observations	124,711	124,711	124,711	57,645	57,645	57,645
Adjusted *R* ^2^	0.006	0.106	0.117	0.006	0.110	0.125

*Note*: Linear regression model with clustered robust standard errors at the hospital level. All models include financial year and month fixed effects. HRGs: Healthcare Resource Groups. Pre‐COVID‐19 period: April 2015 to January 2020. Specification model (1) does not include any controls; specification model (2) excludes past emergency admissions and secondary diagnosis; the full specification model is presented in (3).

*p < 0.05, ** *p* < 0.01, *** *p* < 0.001.

Table 7 provides the results for the COVID‐19 period. For breast conserving surgery, column (1) suggests again that differences in waiting times by deprivation in Table A9 are not statistically significant. In line with our main results in Table 2, we find that patients in the most deprived quintile wait longer by 0.8 days in column (2) and 0.6 days in column (3). For mastectomy, there are no significant differences in waiting times between the least deprived and other groups in column (1). Patients in the most income deprived quintile wait one additional day in column (2) and 0.7 days in column (3). Therefore, waiting time inequalities by income deprivation relate to both breast conserving surgery and mastectomy. When we restrict the analysis by procedure to patients with invasive breast cancer, for breast conserving surgery, patients in the most deprived quintile wait 0.8 days longer in column (3). For mastectomy, patients in the most income deprived quintile wait 0.6 days longer in column (3). The results are therefore qualitatively similar (see Table A13).

**TABLE 7:** COVID‐19: Regression results by type of procedure.

	Breast conserving surgery			Mastectomy		
	(1) Waiting time	(2) Waiting time	(3) Waiting time	(1) Waiting time	(2) Waiting time	(3) Waiting time
Income deprivation (baseline 1 ‐ least deprived)						
2nd Income deprived quintile	0.0906	0.259	0.222	−0.329	−0.0365	−0.0473
3rd Income deprived quintile	0.159	0.328*	0.233	−0.0350	0.306	0.190
4th Income deprived quintile	0.107	0.669***	0.502**	−0.397	0.420	0.252
Most income deprived quintile	0.212	0.844***	0.584**	0.0642	0.972***	0.741**
Hospital fixed effects	No	Yes	Yes	No	Yes	Yes
HRGs effects	No	Yes	Yes	No	Yes	Yes
Observations	49,892	49,892	49,892	22,924	22,924	22,924
Adjusted *R* ^2^	0.027	0.159	0.167	0.033	0.157	0.167

*Note*: Linear regression model with clustered robust standard errors at the hospital level. All models include financial year and month fixed effects. HRGs: Healthcare Resource Groups. COVID‐19 period: February 2020 to March 2022. Specification model (1) does not include any controls; specification model (2) excludes past emergency admissions and secondary diagnosis; the full specification model is presented in (3).

*p < 0.05, ** *p* < 0.01, *** *p* < 0.001.

### 7.5 Distance

Distance to hospital could potentially affect how long patients wait. For example, if a slot becomes suddenly available, then patients who live closer to the hospital may be more willing to accept it at short notice. On the other hand, patients who live further away (e.g., in rural areas) may be more used to traveling and therefore willing to travel to the hospital (Moscelli et al., 2016). We therefore run a robustness check where we add as an additional covariate the distance from where the patient resides to the hospital where the patient receives treatment. However, distance is potentially endogenous: longer waiting times can induce patients to choose providers that are located further away (Moscelli et al., 2016), increasing the distance traveled. Therefore, we did not include distance in our initial regression analysis.

We compute straight‐line distances using geographical coordinates between the centroid of patients' LSOA of residence and the location of the hospital where the patient is treated. Distance bands were created based on the percentiles 25, 50, 75, and 95 of the distance distribution in the pre‐COVID‐19 and COVID‐19 sample, and use the band with the shortest distance as the reference group. For the pre‐COVID‐19 sample the bands are: less or equal than 5, 5–10 km, 10–20 km, 20–38 km, and above 38 km. For the COVID‐19 sample the bands are: less or equal than 5, 5–11 km, 11–21 km, 21–50 km, and above 50 km. Tables A14 and A15 in the Appendix present the results for the pre‐COVID‐19 and COVID‐19 periods, respectively, for both full specification model (column (1)) and when excluding hospital fixed effects (column (2)).

The results are in line with the main analysis. Specifically, in the pre‐COVID‐19 period, we find that there are no statistically significant differences in inpatient waiting times across deprivation quintiles. In the COVID‐19 period, patients in the most income deprived quintile wait 0.6 days longer relative to the least deprived (column (1) of Table A14), which is very similar to the result provided in Table 2. Instead, there is no association between deprivation and waiting times when not controlling for hospital fixed effects (column (2) of Table A15), which is in line with Table 3.

## 8 DISCUSSION

In this paper we have investigated socioeconomic inequalities in waiting times for surgery for breast cancer patients both before and after the COVID‐19 pandemic. In the pre‐COVID‐19 period (April 2015–January 2020), we find that, conditional on patients attending a given hospital and controlling for a range of patients characteristics (including the number and type of comorbidities and past emergency admissions), income deprivation is not associated with inpatient waiting time for surgery. In contrast, in the COVID‐19 period (February 2020–March 2022), we find that patients living in the most deprived areas have longer waiting times by 0.7 days or 2.9% at the sample mean. The effect is smaller for patients living in the 3rd and 4th most income deprived areas who wait 0.2 and 0.4 days longer, respectively.

During the pandemic, hospitals had to suspend surgeries during the lockdowns and this resulted in a reduction in breast cancer surgeries by 29% in 2020/21. Such reductions were evenly distributed across socioeconomic deprivation groups. 14.8% of surgeries were performed for the most deprived quintile pre‐COVID‐19, which reduced to 14.3% in the COVID‐19 period. The results are in line with existing literature, which finds disparities in volume amongst income deprived across a range of elective surgeries (e.g., Cookson et al., 2015) and cancer surgeries (Raine et al., 2010). Volume of surgery resumed to pre‐pandemic levels in 2021/22. Initially, fewer cancer patients were added to the waiting list, and waiting times reduced from 20.6 days in 2019/20 to 18.6 days in 2020/21, and then increased by 3.6 days (to 22.2 days) in 2021/22. We provide evidence that some waiting time inequalities arose during the pandemic and that differences in waiting times across deprivation quintiles are less than one day. One possible explanation for this small difference in waiting times is that during the pandemic patients had to be tested for COVID‐19 and the prevalence of patients testing positive differs by socioeconomic status (Morrissey et al., 2021) and is higher among those living in more deprived areas (e.g., due to the nature of the type of work and other factors). Testing positive for COVID‐19 would result in a delay in surgery by several weeks to allow patients to recover fully and due to concerns of additional complication risk from general anesthesia.

Our findings for the pre‐COVID‐19 period are consistent with findings by Saito et al. (2021) on colon cancer in England, which found no differences in waiting times by income deprivation in 2010–2013. In this study, waiting times for colon cancer were on average 36 days, which is higher than in our sample (about 20 days). Bosque‐Mercader et al. (2023) also find no differences in waiting times by socioeconomic status for patients with breast cancer in Spain when waiting times were on average 21 days in 2015–2019.

Waiting times for breast cancer surgery in England are lower than for other elective surgeries such as hip and knee replacements, coronary bypass and angioplasty. Laudicella et al. (2012) show for hip replacement that patients living in the most income deprived areas waited 7% longer than those in the least deprived areas in 2001 in England. Given an average waiting time of 250 days, this amounts to a difference of 17 days. Similarly, Kasteridis et al. (2023) show a difference of 20 days between hip replacement patients living in the most and least deprived areas in 2020/21 when average waiting times were 220 days. Moscelli et al. (2018) show that for coronary bypass patients living in the most income deprived quintile waited 29% longer relative to least deprived quintile in 2002, and this reduced to 16% in 2006 and further to 9% in 2010. Given that waiting times for coronary bypass were on average 153 days in 2002, 66 days in 2006, and 50 days in 2010, the difference in waiting times amounted respectively to 44 days in 2002, 11 days in 2006 and 5 days in 2010. The study therefore suggests that differences in waiting times by income deprivation tend to reduce when the average waiting time reduces both in absolute values (in days) and in percentage terms. We therefore conclude that, relative to the studies on hip replacement and coronary bypass in England, the difference in waiting times for breast cancer are smaller both in days (less than one day) and in percentage terms (less than 3%).

Our findings on the association between waiting times and income deprivation are robust to controlling for the number of comorbidities and past emergency admissions, though the effect of deprivation on waiting time is slightly more pronounced (but still less than a day) when not controlling for these variables. This is because patients living in more deprived areas have a higher prevalence of comorbidities and had more past emergency admissions in the year preceding the surgery. But because more comorbidities and more past emergency admissions are associated with a longer waiting time of up to a couple of days, patients living in more deprived areas tend to wait more on average. We conjecture that patients with more complex health backgrounds may require more contacts with the health system (e.g., arrange different appointments across specialties) and additional checks that they are fit for surgery, therefore translating into a longer waiting time.

Our results highlight the need for targeted interventions to mitigate inequalities that emerged during COVID‐19, such as policies that reduce barriers to timely surgery for patients living in more deprived areas. Furthermore, improved coordination is essential for patients with complex health histories. Creating an integrated care pathway could smooth pre‐surgical processes and minimise delays caused by additional appointments and checks.

Future research could explore the waiting time for outpatient appointments, from referral to specialist visit, as these may constitute a substantial proportion of total time waited. Additionally, extending the analysis to include more recent financial years would be valuable in determining whether the inequalities identified during the COVID‐19 period have persisted over time.

Our study had some limitations. First, patients may differ in the stage of cancer and this could correlate both with waiting time and socioeconomic status. When patients are added to the waiting list in England they are classified as P2 (wait less than a month), and this includes DCIS. The vast majority of breast cancers are operable at diagnosis and will take a long time to become inoperable. Therefore, even if patients with low socioeconomic status tend to have later stage cancers (larger cancer or more nodal involvement) that should not have a large impact on how long they wait for surgery if their cancer is operable. Second, we do not account for possible variations in cancer detection rates across income deprivation groups. Generally, evidence suggests that cancer is more likely to be detected earlier in less disadvantaged patient groups (Booth et al., 2009; Corner & Brindle, 2011), and that breast cancer admissions among patients from more deprived areas are more likely to be emergency admissions and to have a mastectomy as opposed to breast‐conserving surgery (Downing et al., 2007; Raine et al., 2010). As we cannot observe patients who remain undiagnosed, there may be a source of selection bias, implying that we underestimate the degree of socioeconomic inequality in access to breast cancer treatment.

## CONFLICT OF INTEREST STATEMENT

The authors declare no conflict of interest statement.

## Supporting information

Supporting Information S1

## Data Availability

The data that support the findings of this study are available from England. Restrictions apply to the availability of the following datasets that were used under licence for this study. This work uses data provided by patients and collected by the NHS as part of their care and support. The HES are copyright ©2015/16–2021/22, NHS England. Re‐used with the permission of NHS England. All rights reserved.

## ENDNOTES

^1^

Statistics » Cancer Waiting Times (england.nhs.uk).

^2^
CCGs were clinically‐led statutory NHS bodies responsible for the planning and commissioning of health care services for their local area from 2013 to 2022.

^3^
A spell may include more than one Finished Consultant Episode (FCE) defined as a period of care under the same consultant (senior doctor).

^4^
Therefore, we use HES data for the financial year 2013/14 and 2014/15 to check for patients who had a breast cancer diagnosis in the financial year 2015/16 and 2016/17.

^5^
LSOAs are designed to be consistent in population size with an average of approximately 1500 inhabitants.

^6^
For waiting times we use the Interquartile Range rule method. An outlier is any observation that is more than three times the Interquartile Range (difference between the third and first quartile) above the third quartile, or below the first quartile. As for the length of stay (LOS), and given its highly skewed distribution, we exclude all observations with a LOS above the 99th percentile.

^7^
To identify outliers we apply the same methods as in the pre‐COVID‐19 sample.

^8^
Table A1 provides the same regression results in Table 2 with standard errors. In Table A2, we provide the results when we use a more flexible time trend (month‐year fixed effects). The results are very similar to our main approach.

^9^
Table A7, provides the results when we use a more flexible time trend (month‐year fixed effects). The results are very similar to our main approach. This is also the case for Tables A8, A10, and A12 which replicate Tables 5, 6, 7, respectively. Again, the results are very similar to our main approach.
