# Supplementary material for: Socioeconomic inequalities in waiting times for breast cancer surgery
Source: Health Econ. 2024 Oct 3;34(2):203–24. doi: 10.1002/hec.4906 (PMC11700930; doi:10.1002/hec.4906)
Supplement: Supplementary file 1 — Supporting Information S1 [file HEC-34-203-s001.docx]

**Appendix**

**Figure A1. Typical care pathway for cancer treatment.**


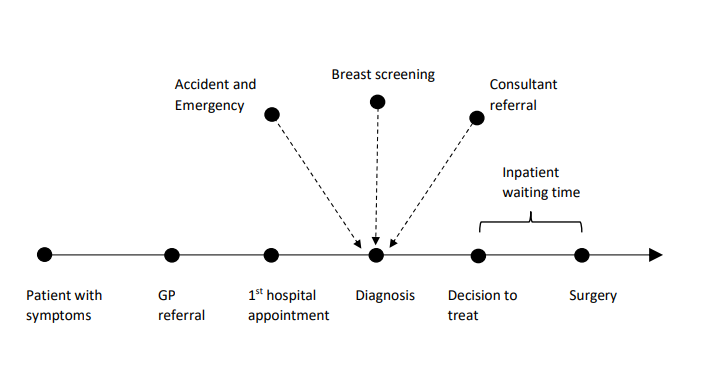


Source: Adapted from Department of Health (2000).

**Table A1. Regression results including HRGs and standard errors.**

|  | (1) | (2) |
| --- | --- | --- |
|  | Waiting time  Pre-COVID-19 | Waiting time  COVID-19 |
| **Income deprivation (baseline 1 - least deprived)** |  |  |
| 2nd income deprived quintile | 0.0703 | 0.137 |
|  | (0.0849) | (1.24) |
| 3rd income deprived quintile | 0.0258 | 0.238* |
|  | (0.0948) | (2.23) |
| 4th income deprived quintile | 0.0855 | 0.430** |
|  | (0.0909) | (3.00) |
| Most income deprived quintile | 0.114 | 0.657*** |
|  | (0.0938) | (4.65) |
| **Age groups (baseline <45)** |  |  |
| [45,50[ | 0.429** | -0.429* |
|  | (0.140) | (-2.06) |
| [50,55[ | 0.640*** | 0.185 |
|  | (0.139) | (0.95) |
| [55,60[ | 0.793*** | 0.328 |
|  | (0.133) | (1.69) |
| [60,65[ | 0.980*** | 0.428* |
|  | (0.154) | (2.20) |
| [65,70[ | 0.981*** | 0.555* |
|  | (0.150) | (2.61) |
| [70,75[ | 0.988*** | 0.522* |
|  | (0.167) | (2.22) |
| [75,80[ | 0.986*** | 0.309 |
|  | (0.188) | (1.26) |
| [80,102] | 1.695*** | 1.582*** |
|  | (0.213) | (5.76) |
| **Ethnicity (baseline=White)** |  |  |
| Mixed | -0.125 | -0.846 |
|  | (0.317) | (-1.78) |
| Asian | -0.463* | -0.152 |
|  | (0.180) | (-0.61) |
| Black | 0.0742 | -0.343 |
|  | (0.253) | (-1.35) |
| Others | -0.140 | -0.145 |
|  | (0.196) | (-0.50) |
| Missing | -0.279* | -0.345* |
|  | (0.129) | (-2.14) |
| **Mastectomy (B27)** | -0.0555 | -0.0366 |
|  | (0.100) | (-0.26) |
| **Invasive breast cancer (C50)** | -0.767*** | -0.781*** |
|  | (0.0986) | (-4.91) |
| C77 - Secondary and unspecified malignant neoplasm of lymph nodes | -0.316** | -0.210 |
|  | (0.103) | (-1.50) |
| D05 - Ductal carcinoma in situ of breast | -0.493** | -0.309 |
|  | (0.166) | (-1.08) |
| Z85 - Personal history of malignant neoplasm | -0.506*** | -0.511** |
|  | (0.131) | (-3.36) |
| Z80 - Family history of malignant neoplasm | -0.221 | -0.274 |
|  | (0.134) | (-1.44) |
| Z86 - Personal history of certain other diseases | 0.000803 | -0.128 |
|  | (0.0834) | (-0.89) |
| Z87 - Personal history of other diseases and conditions | -0.298 | -0.104 |
|  | (0.180) | (-0.46) |
| Z88 - Personal history of allergy to drugs, medicaments and biological substances | -0.0722 | -0.0548 |
|  | (0.108) | (-0.43) |
| Z90 - Acquired absence of organs, not elsewhere classified | -0.178 | -0.260 |
|  | (0.112) | (-1.77) |
| Z92 - Personal history of medical treatment | 2.631*** | 2.310*** |
|  | (0.165) | (11.04) |
| Z96 - Presence of other functional implants | -0.146 | -0.0339 |
|  | (0.152) | (-0.18) |
| Z11- Encounter for screening for infectious and parasitic diseases |  | 0.286 |
|  |  | (0.67) |
| E03 - Other hypothyroidism | -0.0436 | -0.120 |
|  | (0.1000) | (-0.70) |
| E11 - Non-insulin-dependent diabetes mellitus | 0.0302 | 0.519** |
|  | (0.105) | (2.67) |
| E66 - Obesity due to excess calories | 0.0225 | 0.173 |
|  | (0.101) | (1.11) |
| E78 - Disorders of lipoprotein metabolism and other lipidaemis | -0.167 | 0.135 |
|  | (0.133) | (0.58) |
| I10 - Essential (primary) hypertension | 0.00270 | 0.0667 |
|  | (0.0695) | (0.56) |
| I25 - Chronic ischemic heart disease | 0.348 | 0.382 |
|  | (0.183) | (1.40) |
| I48 - Atrial fibrillation and flutter | -0.466* | -0.425 |
|  | (0.184) | (-1.34) |
| J44 - Other chronic obstructive pulmonary disease | 0.559*** | 0.488* |
|  | (0.147) | (2.08) |
| J45 - Predominantly allergic asthma | -0.122 | -0.0555 |
|  | (0.0927) | (-0.36) |
| K21 - Gastro-oesophageal reflux disease | -0.0764 | -0.108 |
|  | (0.122) | (-0.74) |
| M13 - Other arthritis | -0.314 | 0.235 |
|  | (0.176) | (0.80) |
| M19 - Other arthrosis | -0.0198 | -0.129 |
|  | (0.137) | (-0.75) |
| F17 - Mental and behavioural disorders due to use of tobacco | -0.173 | 0.0701 |
|  | (0.104) | (0.41) |
| F32 - Depressive episode | -0.00392 | 0.155 |
|  | (0.115) | (0.76) |
| F41 - Other anxiety disorders | -0.325** | -0.233 |
|  | (0.120) | (-1.47) |
| Z13 - Special screening examination for other diseases and disorders | 1.388 | 2.180** |
|  | (1.101) | (2.68) |
| Other diagnoses | -0.123 | 0.137 |
|  | (0.0846) | (0.98) |
| **Secondary Diagnosis (baseline=0)** |  |  |
| One diagnosis | 0.435*** | -0.117 |
|  | (0.104) | (-0.59) |
| Two diagnoses | 0.687*** | 0.278 |
|  | (0.122) | (1.22) |
| Three diagnoses | 0.885*** | 0.275 |
|  | (0.159) | (1.01) |
| Four diagnoses | 0.981*** | 0.435 |
|  | (0.176) | (1.46) |
| Five diagnoses | 0.904*** | 0.440 |
|  | (0.234) | (1.23) |
| More than six diagnoses | 1.035*** | 0.573 |
|  | (0.253) | (1.46) |
| **Past emergency admissions (baseline=0)** |  |  |
| [1,2] | 2.153*** | 1.545*** |
|  | (0.159) | (8.06) |
| Above 2 | 3.785*** | 1.474** |
|  | (0.342) | (3.34) |
| **HRGs (baseline=JA32: Unilateral Excision of Breast with Immediate Pedicled Myocutaneous Flap Reconstruction)** |  |  |
| JA12: Malignant Breast Disorders with Interventions | -3.165 | 25.08*** |
|  | (2.311) | (52.55) |
| JA20: Unilateral Major Breast Procedures | -1.312*** | -1.505*** |
|  | (0.242) | (-6.37) |
| JA21: Bilateral Major Breast Procedures | -0.180 | 0.0573 |
|  | (0.264) | (0.18) |
| JA30: Unilateral Delayed Pedicled Myocutaneous Breast Reconstruction | -1.290* | -0.737 |
|  | (0.627) | (-0.66) |
| JA31: Bilateral Delayed Pedicled Myocutaneous Breast Reconstruction | 0.189 | 2.694 |
|  | (3.757) | (0.90) |
| JA33: Bilateral Excision of Breast with Immediate Pedicled Myocutaneous Flap Reconstruction | 2.062*** | 1.706** |
|  | (0.408) | (2.72) |
| JA34: Unilateral Delayed Free Perforated Flap Breast Reconstruction | -4.980 | 0.234 |
|  | (3.207) | (0.06) |
| JA36: Unilateral Excision of Breast with Immediate Free Perforator Flap Reconstruction | 4.011*** | 4.233** |
|  | (1.182) | (2.75) |
| JA37: Bilateral Excision of Breast with Immediate Free Perforator Flap Reconstruction | 5.996*** | 6.152** |
|  | (1.412) | (2.75) |
| JA38: Unilateral Major Breast Procedures with Lymph Node Clearance | -0.0423 | -0.565 |
|  | (0.257) | (-1.97) |
| JA39: Bilateral Major Breast Procedures with Lymph Node Clearance | 1.505** | 1.550* |
|  | (0.484) | (2.27) |
| JA40: Unilateral Therapeutic Mammoplasty | -0.279 | -0.960* |
|  | (0.382) | (-2.33) |
| JA41: Bilateral Therapeutic Mammoplasty | 0.578 | 0.798 |
|  | (0.444) | (1.85) |
| JA42: Bilateral Intermediate Breast Procedures | -1.565 | 1.402 |
|  | (1.651) | (0.36) |
| JA43: Unilateral Intermediate Breast Procedures | -1.239** | -1.399** |
|  | (0.371) | (-3.32) |
| JA44: Bilateral Minor Breast Procedures | -14.68*** |  |
|  | (0.351) |  |
| JA45: Unilateral Minor Breast Procedures | -3.843 | -0.382 |
|  | (2.078) | (-0.22) |
| **Financial Year (baseline 2015/16)** |  |  |
| 2016/17 | 0.0897 |  |
|  | (0.294) |  |
| 2017/18 | -0.224 |  |
|  | (0.316) |  |
| 2018/19 | 0.631* |  |
|  | (0.295) |  |
| 2019/20 | 0.951** |  |
|  | (0.345) |  |
| **Financial Year (baseline 2019/20)** |  |  |
| 2020/21 |  | 0.317 |
|  |  | (0.72) |
| 2021/22 |  | 2.642*** |
|  |  | (6.40) |
| **Month (baseline April)** |  |  |
| January | 1.579*** | 3.769** |
|  | (0.207) | (9.05) |
| February | -0.843*** | 1.839*** |
|  | (0.177) | (4.17) |
| March | -0.563** | 1.342** |
|  | (0.202) | (3.31) |
| May | -0.102 | 1**.**086** |
|  | (0.156) | (3.04) |
| June | 0.399* | 1.898*** |
|  | (0.195) | (5.33) |
| July | -0.0523 | 2.192*** |
|  | (0.194) | (6.26) |
| August | 0.290 | 2.618*** |
|  | (0.202) | (6.93) |
| September | 1.354*** | 2.883*** |
|  | (0.182) | (7.26) |
| October | 0.252 | 1.706*** |
|  | (0.192) | (4.27) |
| November | 0.105 | 1.742*** |
|  | (0.213) | (4.73) |
| December | -0.751*** | 1.763*** |
|  | (0.206) | (4.33) |
| Constant | 3.991*** | 7.193*** |
|  | (0.485) | (10.02) |
| Hospital fixed effects | Yes | Yes |
| Observations | 182,356 | 72,816 |
| Adjusted R2 | 0.118 | 0.164 |
| **p*< 0.05, ***p*< 0.01, ****p*< 0.001. t statistics in parentheses. |  |  |
| Linear regression model with clustered robust standard errors at the hospital level. | |  |

Pre-COVID-19 period: April 2015 to January 2020. COVID-19 period: February 2020 to March 2022.

**Table A2. Regression results with flexible time trends**

|  | (1)  Waiting time  Pre-COVID-19 | (2)  Waiting time  COVID-19 |
| --- | --- | --- |
| **Income deprivation (baseline 1 - least deprived)** |  |  |
| 2nd income deprived quintile | 0.0690 | 0.142 |
| 3rd income deprived quintile | 0.0241 | 0.255* |
| 4th income deprived quintile | 0.0859 | 0.441** |
| Most income deprived quintile | 0.118 | 0.667*** |
| Hospital fixed effects | Yes | Yes |
| HRGs effects | Yes | Yes |
| Observations | 182,356 | 72,816 |
| Adjusted *R*^2^ | 0.119 | 0.167 |
| Notes. * *p* < 0.05, ** *p* < 0.01, *** *p* < 0.001. Linear regression model with clustered robust standard errors at the hospital level. All models include financial month-year fixed effects. We also control for age, ethnicity, type of diagnosis, type of procedure, secondary diagnosis, and past emergency admissions. HRGs: Healthcare Resource Groups. Pre-COVID-19 period: April 2015 to January 2020. COVID-19 period: February 2020 to March 2022. | | |

**Table A3. Pre-COVID-19: Descriptive statistics (mean) by socioeconomic status**

|  | Least income deprived quintile | 2nd income deprived quintile | 3rd income deprived quintile | 4th income deprived quintile | Most income deprived quintile |
| --- | --- | --- | --- | --- | --- |
| Waiting time (days) | 20.105 | 20.103 | 20.133 | 19.973 | 19.917 |
| Mastectomy | 0.301 | 0.312 | 0.312 | 0.324 | 0.343 |
| **Primary diagnosis** |  |  |  |  |  |
| Invasive breast cancer (C50) | 0.874 | 0.878 | 0.884 | 0.883 | 0.887 |
| Ductal carcinoma in situ of breast (D05) | 0.126 | 0.122 | 0.116 | 0.117 | 0.113 |
| **Age (years)** | 62.187 | 61.989 | 61.584 | 60.561 | 59.735 |
| [16,44] | 0.073 | 0.077 | 0.088 | 0.104 | 0.116 |
| [45,49] | 0.092 | 0.091 | 0.094 | 0.102 | 0.109 |
| [50,54] | 0.132 | 0.133 | 0.132 | 0.139 | 0.142 |
| [55,59] | 0.122 | 0.123 | 0.123 | 0.125 | 0.132 |
| [60,64] | 0.127 | 0.129 | 0.128 | 0.127 | 0.131 |
| [65,69] | 0.160 | 0.156 | 0.152 | 0.147 | 0.135 |
| [70,74] | 0.127 | 0.125 | 0.121 | 0.109 | 0.098 |
| [75,79] | 0.083 | 0.084 | 0.078 | 0.071 | 0.068 |
| [80,102] | 0.085 | 0.083 | 0.085 | 0.077 | 0.069 |
| **Ethnicity** |  |  |  |  |  |
| White | 0.823 | 0.829 | 0.807 | 0.774 | 0.741 |
| Mixed | 0.003 | 0.004 | 0.006 | 0.006 | 0.008 |
| Asian | 0.016 | 0.018 | 0.033 | 0.048 | 0.062 |
| Black | 0.004 | 0.007 | 0.015 | 0.030 | 0.041 |
| Others | 0.014 | 0.013 | 0.016 | 0.019 | 0.023 |
| Missing | 0.139 | 0.129 | 0.124 | 0.122 | 0.126 |
| **Secondary diagnosis/Comorbidities** |  |  |  |  |  |
| C77 - Secondary and unspecified malignant neoplasm of lymph nodes | 0.179 | 0.193 | 0.195 | 0.203 | 0.215 |
| D05 - Ductal carcinoma in situ of breast | 0.037 | 0.044 | 0.050 | 0.042 | 0.034 |
| Z85 - Personal history of malignant neoplasm | 0.068 | 0.067 | 0.065 | 0.060 | 0.059 |
| Z80 - Family history of malignant neoplasm | 0.075 | 0.075 | 0.070 | 0.069 | 0.067 |
| Z86 - Personal history of certain other diseases | 0.164 | 0.173 | 0.183 | 0.192 | 0.205 |
| Z87 - Personal history of other diseases and conditions | 0.033 | 0.033 | 0.033 | 0.033 | 0.035 |
| Z88 - Personal history of allergy to drugs, medicaments and biological substances | 0.111 | 0.106 | 0.107 | 0.108 | 0.113 |
| Z90 - Acquired absence of organs, not elsewhere classified | 0.070 | 0.068 | 0.066 | 0.064 | 0.067 |
| Z92 - Personal history of medical treatment | 0.123 | 0.124 | 0.124 | 0.131 | 0.146 |
| Z96 - Presence of other functional implants | 0.037 | 0.040 | 0.037 | 0.037 | 0.034 |
| E03 - Other hypothyroidism | 0.071 | 0.072 | 0.077 | 0.076 | 0.076 |
| E11 - Non-insulin-dependent diabetes mellitus | 0.051 | 0.065 | 0.080 | 0.092 | 0.121 |
| E66 - Obesity due to excess calories | 0.094 | 0.107 | 0.121 | 0.133 | 0.150 |
| E78 - Disorders of lipoprotein metabolism and other lipidaemis | 0.040 | 0.043 | 0.051 | 0.062 | 0.076 |
| I10 - Essential (primary) hypertension | 0.260 | 0.270 | 0.287 | 0.294 | 0.323 |
| I25 - Chronic ischemic heart disease | 0.020 | 0.023 | 0.028 | 0.031 | 0.038 |
| I48 - Atrial fibrillation and flutter | 0.031 | 0.030 | 0.030 | 0.030 | 0.028 |
| J44 - Other chronic obstructive pulmonary disease | 0.018 | 0.024 | 0.029 | 0.041 | 0.062 |
| J45 - Predominantly allergic asthma | 0.090 | 0.094 | 0.097 | 0.100 | 0.114 |
| K21 - Gastro-oesophageal reflux disease | 0.048 | 0.052 | 0.056 | 0.061 | 0.073 |
| M13 - Other arthritis | 0.033 | 0.031 | 0.034 | 0.037 | 0.046 |
| M19 - Other arthrosis | 0.035 | 0.039 | 0.039 | 0.044 | 0.055 |
| F17 - Mental and behavioural disorders due to use of tobacco | 0.051 | 0.067 | 0.086 | 0.114 | 0.165 |
| F32 - Depressive episode | 0.042 | 0.051 | 0.056 | 0.064 | 0.084 |
| F41 - Other anxiety disorders | 0.049 | 0.055 | 0.058 | 0.065 | 0.078 |
| Z13 - Special screening examination for other diseases and disorders | 0.034 | 0.024 | 0.031 | 0.038 | 0.054 |
| Other diagnoses | 0.448 | 0.463 | 0.474 | 0.491 | 0.524 |
| **Number of secondary diagnoses/Comorbidities** |  |  |  |  |  |
| None | 0.176 | 0.159 | 0.148 | 0.131 | 0.102 |
| One diagnosis | 0.205 | 0.200 | 0.186 | 0.179 | 0.156 |
| Two diagnoses | 0.184 | 0.178 | 0.179 | 0.176 | 0.163 |
| Three diagnoses | 0.139 | 0.144 | 0.141 | 0.141 | 0.147 |
| Four diagnoses | 0.100 | 0.105 | 0.111 | 0.115 | 0.119 |
| Five diagnoses | 0.068 | 0.071 | 0.077 | 0.081 | 0.092 |
| More than six diagnoses | 0.128 | 0.143 | 0.158 | 0.178 | 0.221 |
| **Past emergency admissions (< 365 days)** |  |  |  |  |  |
| None | 0.905 | 0.899 | 0.889 | 0.874 | 0.856 |
| Between one and two | 0.084 | 0.088 | 0.098 | 0.108 | 0.123 |
| More than 2 | 0.011 | 0.013 | 0.014 | 0.018 | 0.021 |
| Notes: Pre-COVID-19 period: April 2015 to January 2020. | | | | | |

**Table A4. COVID-19: Descriptive statistics (mean) by socioeconomic status**

|  | Least income deprived quintile | 2nd income deprived quintile | 3rd income deprived quintile | 4th income deprived quintile | Most income deprived quintile |
| --- | --- | --- | --- | --- | --- |
| Waiting time (days) | 20.552 | 20.544 | 20.653 | 20.560 | 20.723 |
| Mastectomy | 0.298 | 0.308 | 0.318 | 0.320 | 0.343 |
| **Primary diagnosis** |  |  |  |  |  |
| Invasive breast cancer (C50) | 0.879 | 0.886 | 0.887 | 0.887 | 0.889 |
| Ductal carcinoma in situ of breast (D05) | 0.121 | 0.114 | 0.113 | 0.113 | 0.111 |
| **Age (years)** | 62.525 | 62.352 | 61.668 | 60.525 | 59.410 |
| [16,44] | 0.075 | 0.080 | 0.097 | 0.110 | 0.140 |
| [45,49] | 0.074 | 0.071 | 0.076 | 0.084 | 0.091 |
| [50,54] | 0.132 | 0.133 | 0.126 | 0.136 | 0.133 |
| [55,59] | 0.135 | 0.132 | 0.133 | 0.143 | 0.136 |
| [60,64] | 0.131 | 0.137 | 0.137 | 0.134 | 0.138 |
| [65,69] | 0.141 | 0.143 | 0.144 | 0.136 | 0.130 |
| [70,74] | 0.126 | 0.124 | 0.115 | 0.104 | 0.094 |
| [75,79] | 0.099 | 0.088 | 0.087 | 0.079 | 0.069 |
| [80,102] | 0.087 | 0.092 | 0.085 | 0.072 | 0.068 |
| **Ethnicity** |  |  |  |  |  |
| White | 0.771 | 0.772 | 0.754 | 0.715 | 0.692 |
| Mixed | 0.004 | 0.005 | 0.007 | 0.009 | 0.010 |
| Asian | 0.018 | 0.023 | 0.033 | 0.049 | 0.069 |
| Black | 0.004 | 0.008 | 0.015 | 0.031 | 0.045 |
| Others | 0.018 | 0.014 | 0.020 | 0.026 | 0.026 |
| Missing | 0.185 | 0.179 | 0.172 | 0.170 | 0.158 |
| **Secondary diagnosis/Comorbidities** |  |  |  |  |  |
| C77 - Secondary and unspecified malignant neoplasm of lymph nodes | 0.187 | 0.192 | 0.205 | 0.203 | 0.223 |
| D05 - Ductal carcinoma in situ of breast | 0.054 | 0.064 | 0.061 | 0.057 | 0.051 |
| Z85 - Personal history of malignant neoplasm | 0.078 | 0.079 | 0.080 | 0.074 | 0.068 |
| Z80 - Family history of malignant neoplasm | 0.097 | 0.093 | 0.089 | 0.093 | 0.089 |
| Z86 - Personal history of certain other diseases | 0.208 | 0.218 | 0.221 | 0.226 | 0.245 |
| Z87 - Personal history of other diseases and conditions | 0.056 | 0.051 | 0.053 | 0.050 | 0.061 |
| Z88 - Personal history of allergy to drugs, medicaments and biological substances | 0.134 | 0.131 | 0.139 | 0.137 | 0.139 |
| Z90 - Acquired absence of organs, not elsewhere classified | 0.098 | 0.097 | 0.093 | 0.092 | 0.096 |
| Z92 - Personal history of medical treatment | 0.152 | 0.150 | 0.155 | 0.165 | 0.177 |
| Z96 - Presence of other functional implants | 0.048 | 0.050 | 0.048 | 0.040 | 0.045 |
| Z11- Encounter for screening for infectious and parasitic diseases | 0.026 | 0.031 | 0.032 | 0.034 | 0.035 |
| E03 - Other hypothyroidism | 0.075 | 0.079 | 0.081 | 0.083 | 0.078 |
| E11 - Non-insulin-dependent diabetes mellitus | 0.057 | 0.065 | 0.077 | 0.093 | 0.122 |
| E66 - Obesity due to excess calories | 0.130 | 0.152 | 0.168 | 0.193 | 0.212 |
| E78 - Disorders of lipoprotein metabolism and other lipidaemis | 0.051 | 0.056 | 0.062 | 0.073 | 0.083 |
| I10 - Essential (primary) hypertension | 0.252 | 0.267 | 0.281 | 0.294 | 0.313 |
| I25 - Chronic ischemic heart disease | 0.022 | 0.023 | 0.027 | 0.029 | 0.040 |
| I48 - Atrial fibrillation and flutter | 0.034 | 0.034 | 0.034 | 0.029 | 0.027 |
| J44 - Other chronic obstructive pulmonary disease | 0.018 | 0.026 | 0.033 | 0.039 | 0.062 |
| J45 - Predominantly allergic asthma | 0.097 | 0.100 | 0.100 | 0.108 | 0.117 |
| K21 - Gastro-oesophageal reflux disease | 0.063 | 0.070 | 0.077 | 0.078 | 0.093 |
| M13 - Other arthritis | 0.028 | 0.031 | 0.031 | 0.030 | 0.037 |
| M19 - Other arthrosis | 0.051 | 0.050 | 0.053 | 0.057 | 0.064 |
| F17 - Mental and behavioural disorders due to use of tobacco | 0.043 | 0.059 | 0.076 | 0.104 | 0.151 |
| F32 - Depressive episode | 0.054 | 0.062 | 0.069 | 0.080 | 0.101 |
| F41 - Other anxiety disorders | 0.072 | 0.084 | 0.085 | 0.095 | 0.114 |
| Z13 - Special screening examination for other diseases and disorders | 0.031 | 0.023 | 0.024 | 0.031 | 0.044 |
| Other diagnoses | 0.533 | 0.547 | 0.561 | 0.579 | 0.607 |
| **Number of secondary diagnoses/Comorbidities** |  |  |  |  |  |
| None | 0.137 | 0.122 | 0.113 | 0.103 | 0.085 |
| One diagnosis | 0.171 | 0.163 | 0.151 | 0.142 | 0.119 |
| Two diagnoses | 0.163 | 0.161 | 0.157 | 0.146 | 0.140 |
| Three diagnoses | 0.136 | 0.139 | 0.143 | 0.140 | 0.133 |
| Four diagnoses | 0.109 | 0.117 | 0.112 | 0.121 | 0.122 |
| Five diagnoses | 0.081 | 0.087 | 0.092 | 0.097 | 0.094 |
| More than six diagnoses | 0.203 | 0.211 | 0.232 | 0.252 | 0.306 |
| **Past emergency admissions (< 365 days)** |  |  |  |  |  |
| None | 0.904 | 0.897 | 0.880 | 0.872 | 0.851 |
| Between one and two | 0.087 | 0.091 | 0.106 | 0.111 | 0.128 |
| More than 2 | 0.009 | 0.012 | 0.014 | 0.016 | 0.022 |
| Notes: COVID-19 period: February 2020 to March 2022. | | | | | |

**Table A5. Regression results for pre- and COVID-19 by adding covariates sequentially.**

|  | **Pre-COVID-19** | | | **COVID-19** | | |
| --- | --- | --- | --- | --- | --- | --- |
|  | (1)  Waiting time | (2)  Waiting time | (3)  Waiting time | (1)  Waiting time | (2)  Waiting time | (3)  Waiting time |
| **Income deprivation (baseline 1 - least deprived)** |  |  |  |  |  |  |
| 2nd income deprived quintile | -0.00229 | 0.113 | 0.0703 | -0.0275 | 0.172 | 0.137 |
| 3rd income deprived quintile | 0.0208 | 0.101 | 0.0258 | 0.120 | 0.339** | 0.238* |
| 4th income deprived quintile | -0.123 | 0.223* | 0.0855 | -0.0238 | 0.598*** | 0.430** |
| Most income deprived quintile | -0.182 | 0.338*** | 0.114 | 0.211 | 0.909*** | 0.657*** |
| Hospital fixed effects | No | Yes | Yes | No | Yes | Yes |
| HRGs effects | No | Yes | Yes | No | Yes | Yes |
| Observations | 182,356 | 182,356 | 182,356 | 72,816 | 72,816 | 72,816 |
| Adjusted *R*^2^ | 0.006 | 0.106 | 0.118 | 0.028 | 0.155 | 0.164 |
| Notes: * *p* < 0.05, ** *p* < 0.01, *** *p* < 0.001. Linear regression model with clustered robust standard errors at the hospital level. All models include financial year and month fixed effects. HRGs: Healthcare Resource Groups. Pre-COVID-19 period: April 2015 to January 2020; COVID-19 period: February 2020 to March 2022. Specification model (1) does not include any controls; specification model (2) excludes past emergency admissions and secondary diagnosis; the full specification model is presented in (3). | | | | | | |

**Table A6. Descriptive statistics of waiting times by diagnosis**

|  | **Ductal carcinoma in situ of breast**  **Mean (N) waiting time** | | **Invasive breast cancer**  **Mean (N) waiting time** | |
| --- | --- | --- | --- | --- |
|  | Pre-COVID-19  N=21,802 | COVID-19  N=8,346 | Pre-COVID-19  N=160,554 | COVID-19  N=64,470 |
| **Income deprivation score (quintiles)** |  |  |  |  |
| Least income deprived quintile | 20.48 (5,453) | 21.34 (2,104) | 20.05 (37,685) | 20.44 (15,348) |
| 2nd income deprived quintile | 20.77 (4,993) | 21.29 (1,906) | 20.01 (35,992) | 20.45 (14,817) |
| 3rd income deprived quintile | 20.27 (4,462) | 21.08 (1,718) | 20.11 (33,945) | 20.60 (13,541) |
| 4th income deprived quintile | 20.35 (3,828) | 21.13 (1,467) | 19.92 (28,967) | 20.49 (11,529) |
| Most income deprived quintile | 19.84 (3,066) | 20.46 (1,151) | 19.93 (23,965) | 20.76 (9,235) |

Pre-COVID-19 period: April 2015 to January 2020; COVID-19 period: February 2020 to March 2022.

**Table A7. Pre-COVID-19: Regression results by diagnosis using flexible time trends**

|  | **Ductal carcinoma in situ of breast** | | | **Invasive breast cancer** | | |
| --- | --- | --- | --- | --- | --- | --- |
|  | (1)  Waiting time | (2)  Waiting time | (3)  Waiting time | (1)  Waiting time | (2)  Waiting time | (3)  Waiting time |
| **Income deprivation (baseline 1 - least deprived)** |  |  |  |  |  |  |
| 2nd income deprived quintile | 0.264 | 0.376 | 0.358 | -0.0372 | 0.0778 | 0.0348 |
| 3rd income deprived quintile | -0.260 | -0.214 | -0.255 | 0.0615 | 0.141 | 0.0583 |
| 4th income deprived quintile | -0.154 | 0.267 | 0.201 | -0.112 | 0.221* | 0.0769 |
| Most income deprived quintile | -0.689 | 0.0535 | -0.103 | -0.105 | 0.389*** | 0.155 |
| Hospital fixed effects | No | Yes | Yes | No | Yes | Yes |
| HRGs effects | No | Yes | Yes | No | Yes | Yes |
| Observations | 21,802 | 21,802 | 21,802 | 160,554 | 160,554 | 160,554 |
| Adjusted *R*^2^ | 0.010 | 0.104 | 0.106 | 0.006 | 0.107 | 0.122 |
| Notes: * *p* < 0.05, ** *p* < 0.01, *** *p* < 0.001. Linear regression model with clustered robust standard errors at the hospital level. All models include month-year fixed effects. HRGs: Healthcare Resource Groups. Pre-COVID-19 period: April 2015 to January 2020. Specification model (1) does not include any controls; specification model (2) excludes past emergency admissions and secondary diagnosis; the full specification model is presented in (3). | | | | | | |

**Table A8. COVID-19: Regression results by diagnosis using flexible time trends**

|  | **Ductal carcinoma in situ of breast** | | | **Invasive breast cancer** | | |
| --- | --- | --- | --- | --- | --- | --- |
|  | (1)  Waiting time | (2)  Waiting time | (3)  Waiting time | (1)  Waiting time | (2)  Waiting time | (3)  Waiting time |
| **Income deprivation (baseline 1 - least deprived)** |  |  |  |  |  |  |
| 2nd income deprived quintile | -0.0816 | -0.0980 | -0.134 | -0.0111 | 0.199 | 0.166 |
| 3rd income deprived quintile | -0.191 | 0.126 | 0.0489 | 0.193 | 0.384*** | 0.284* |
| 4th income deprived quintile | -0.223 | 0.283 | 0.145 | 0.0298 | 0.646*** | 0.476** |
| Most income deprived quintile | -0.847 | 0.218 | -0.0107 | 0.366 | 0.992*** | 0.742*** |
| Hospital fixed effects | No | Yes | Yes | No | Yes | Yes |
| HRGs effects | No | Yes | Yes | No | Yes | Yes |
| Observations | 8,346 | 8,346 | 8,346 | 64,470 | 64,470 | 64,470 |
| Adjusted *R*^2^ | 0.029 | 0.156 | 0.159 | 0.033 | 0.160 | 0.169 |
| Notes: * *p* < 0.05, ** *p* < 0.01, *** *p* < 0.001. Linear regression model with clustered robust standard errors at the hospital level. All models include month-year fixed effects. HRGs: Healthcare Resource Groups. COVID-19 period: February 2020 to March 2022. Specification model (1) does not include any controls; specification model (2) excludes past emergency admissions and secondary diagnosis; the full specification model is presented in (3). | | | | | | |

**Table A9. Descriptive statistics of waiting times by type of procedure**

|  | **Breast Conserving Surgery**  **Mean (N) waiting time** | | **Mastectomy**  **Mean (N) waiting time** | |
| --- | --- | --- | --- | --- |
|  | Pre-COVID-19  N=124,711 | COVID-19  N=49,892 | Pre-COVID-19  N=57,645 | COVID-19  N=22,924 |
| **Waiting Times** | 19.72 (124,711) | 20.39 (49,892) | 20.78 (57,645) | 21.05 (22,924) |
| **Income deprivation score (quintiles)** |  |  |  |  |
| Least income deprived quintile | 19.77 (30,172) | 20.27 (12,253) | 20.88 (12,966) | 21.21 (5,199) |
| 2nd income deprived quintile | 19.79 (28,190) | 20.40 (11,574) | 20.80 (12,795) | 20.88 (5,149) |
| 3rd income deprived quintile | 19.77 (26,431) | 20.43 (10,408) | 20.93 (11,976) | 21.13 (4,851) |
| 4th income deprived quintile | 19.66 (26,168) | 20.42 (8,833) | 20.62 (10,627) | 20.85 (4,163) |
| Most income deprived quintile | 19.55 (17,750) | 20.45 (6,824) | 20.62 (9,281) | 21.24 (3,562) |

Pre-COVID-19 period: April 2015 to January 2020; COVID-19 period: February 2020 to March 2022.

**Table A10. Pre-COVID-19: Regression results by type of procedure using flexible time trends**

|  | **Breast conserving surgery** | | | **Mastectomy** | | |
| --- | --- | --- | --- | --- | --- | --- |
|  | (1)  Waiting time | (2)  Waiting time | (3)  Waiting time | (1)  Waiting time | (2)  Waiting time | (3)  Waiting time |
| **Income deprivation (baseline 1 - least deprived)** |  |  |  |  |  |  |
| 2nd income deprived quintile | 0.00724 | 0.117 | 0.0808 | -0.0688 | 0.102 | 0.0460 |
| 3rd income deprived quintile | -0.00497 | 0.0495 | -0.0193 | 0.0248 | 0.165 | 0.0762 |
| 4th income deprived quintile | -0.107 | 0.179 | 0.0561 | -0.236 | 0.323* | 0.155 |
| Most income deprived quintile | -0.213 | 0.269* | 0.0583 | -0.249 | 0.487** | 0.242 |
| Hospital fixed effects | No | Yes | Yes | No | Yes | Yes |
| HRGs effects | No | Yes | Yes | No | Yes | Yes |
| Observations | 124,711 | 124,711 | 124,711 | 57,645 | 57,645 | 57,645 |
| Adjusted *R*^2^ | 0.007 | 0.107 | 0.118 | 0.006 | 0.110 | 0.126 |
| Notes. * *p* < 0.05, ** *p* < 0.01, *** *p* < 0.001. Linear regression model with clustered robust standard errors at the hospital level. All models include month-year fixed effects. HRGs: Healthcare Resource Groups. Pre-COVID-19 period: April 2015 to January 2020. Specification model (1) does not include any controls; specification model (2) excludes past emergency admissions and secondary diagnosis; the full specification model is presented in (3). | | | | | | |

**Table A11. Pre-COVID-19: Regression results for waiting times for invasive breast cancer and type of procedure**

|  | **Invasive breast cancer & Breast conserving surgery** | | | **Invasive breast cancer & Mastectomy** | | |
| --- | --- | --- | --- | --- | --- | --- |
|  | (1)  Waiting time | (2)  Waiting time | (3)  Waiting time | (1)  Waiting time | (2)  Waiting time | (3)  Waiting time |
| **Income deprivation (baseline 1 - least deprived)** |  |  |  |  |  |  |
| 2nd income deprived quintile | -0.00805 | 0.101 | 0.0627 | -0.128 | 0.0310 | -0.0215 |
| 3rd income deprived quintile | 0.0510 | 0.108 | 0.0304 | 0.0454 | 0.175 | 0.0816 |
| 4th income deprived quintile | -0.0678 | 0.210 | 0.0745 | -0.284 | 0.248 | 0.0712 |
| Most income deprived quintile | -0.131 | 0.340** | 0.117 | -0.213 | 0.473** | 0.223 |
| Hospital fixed effects | No | Yes | Yes | No | Yes | Yes |
| HRGs effects | No | Yes | Yes | No | Yes | Yes |
| Observations | 108,191 | 108,191 | 108,191 | 52,363 | 52,363 | 52,363 |
| Adjusted *R*^2^ | 0.006 | 0.108 | 0.120 | 0.005 | 0.108 | 0.126 |
| Notes. * *p* < 0.05, ** *p* < 0.01, *** *p* < 0.001. Linear regression model with clustered robust standard errors at the hospital level. All models include financial year and month fixed effects. HRGs: Healthcare Resource Groups. Pre-COVID-19 period: April 2015 to January 2020. Specification model (1) does not include any controls; specification model (2) excludes past emergency admissions and secondary diagnosis; the full specification model is presented in (3). | | | | | | |

**Table A12. COVID-19: Regression results by type of procedure using flexible time trends**

|  | **Breast conserving surgery** | | | **Mastectomy** | | |
| --- | --- | --- | --- | --- | --- | --- |
|  | (1)  Waiting time | (2)  Waiting time | (3)  Waiting time | (1)  Waiting time | (2)  Waiting time | (3)  Waiting time |
| **Income deprivation (baseline 1 - least deprived)** |  |  |  |  |  |  |
| 2nd income deprived quintile | 0.0954 | 0.258 | 0.222 | -0.314 | -0.0263 | -0.0361 |
| 3rd income deprived quintile | 0.176 | 0.339* | 0.246 | -0.0103 | 0.323 | 0.210 |
| 4th income deprived quintile | 0.122 | 0.674*** | 0.510** | -0.385 | 0.428 | 0.263 |
| Most income deprived quintile | 0.238 | 0.862*** | 0.605*** | 0.0630 | 0.953*** | 0.730** |
| Hospital fixed effects | No | Yes | Yes | No | Yes | Yes |
| HRGs effects | No | Yes | Yes | No | Yes | Yes |
| Observations | 49,892 | 49,892 | 49,892 | 22,924 | 22,924 | 22,924 |
| Adjusted *R*^2^ | 0.030 | 0.162 | 0.170 | 0.037 | 0.160 | 0.170 |
| Notes. * *p* < 0.05, ** *p* < 0.01, *** *p* < 0.001. Linear regression model with clustered robust standard errors at the hospital level. All models include financial year and month fixed effects. HRGs: Healthcare Resource Groups. COVID-19 period: February 2020 to March 2022. Specification model (1) does not include any controls; specification model (2) excludes past emergency admissions and secondary diagnosis; the full specification model is presented in (3). | | | | | | |

**Table A13. COVID-19: Regression results for waiting times for invasive breast cancer and type of procedure**

|  | **Invasive breast cancer & Breast conserving surgery** | | | **Invasive breast cancer & Mastectomy** | | |
| --- | --- | --- | --- | --- | --- | --- |
|  | (1)  Waiting time | (2)  Waiting time | (3)  Waiting time | (1)  Waiting time | (2)  Waiting time | (3)  Waiting time |
| **Income deprivation (baseline 1 - least deprived)** |  |  |  |  |  |  |
| 2nd income deprived quintile | 0.133 | 0.321* | 0.284* | -0.384 | -0.0839 | -0.0949 |
| 3rd income deprived quintile | 0.260 | 0.417** | 0.322* | -0.0757 | 0.219 | 0.1000 |
| 4th income deprived quintile | 0.218 | 0.773*** | 0.601*** | -0.504 | 0.315 | 0.131 |
| Most income deprived quintile | 0.424 | 1.015*** | 0.756*** | 0.0542 | 0.860** | 0.616* |
| Hospital fixed effects | No | Yes | Yes | No | Yes | Yes |
| HRGs effects | No | Yes | Yes | No | Yes | Yes |
| Observations | 43,587 | 43,587 | 43,587 | 20,883 | 20,883 | 20,883 |
| Adjusted *R*^2^ | 0.029 | 0.162 | 0.170 | 0.034 | 0.157 | 0.168 |
| Notes. * *p* < 0.05, ** *p* < 0.01, *** *p* < 0.001. Linear regression model with clustered robust standard errors at the hospital level. All models include financial year and month fixed effects. HRGs: Healthcare Resource Groups. COVID-19 period: February 2020 to March 2022. Specification model (1) does not include any controls; specification model (2) excludes past emergency admissions and secondary diagnosis; the full specification model is presented in (3). | | | | | | |

**Table A14. Pre-COVID-19: Regression results for waiting times including distance**

|  | (1)  Waiting Time | (2)  Waiting Time |
| --- | --- | --- |
| **Income deprivation (baseline 1 - least deprived)** |  |  |
| 2nd income deprived quintile | 0.0757 | 0.00108 |
| 3rd income deprived quintile | 0.0286 | 0.0178 |
| 4th income deprived quintile | 0.0798 | -0.171 |
| Most income deprived quintile | 0.0936 | -0.320 |
| **Distance (baseline <= 5km)** |  |  |
| ]5,10] | -0.0279 | -0.0568 |
| ]10,20] | -0.0954 | -0.0995 |
| ]20,38] | -0.264 | -0.150 |
| Above 38 | -0.570 | -0.506 |
| Hospital fixed effects | Yes | No |
| HRGs effects | Yes | Yes |
| Observations | 182,356 | 182,356 |
| Adjusted *R*^2^ | 0.119 | 0.029 |

Notes: * *p* < 0.05, ** *p* < 0.01, *** *p* < 0.001. Linear regression model with clustered robust standard errors at the hospital level. All models include financial year and month fixed effects. Pre-COVID-19 period: April 2015 to January 2020. The full model specification is presented in (1), i.e., it all covariates (age, ethnicity, secondary diagnosis, past emergency readmissions, HRGs and hospital fixed effects). Model (2) includes all covariates except the hospital fixed effects. Distance bands were created based on the percentiles 25, 50, 75, and 95 of the distance distribution. HRGs: Healthcare Resource Groups. “Distance” is the distance between the patient location and hospital location.

**Table A15. COVID-19: Regression results for waiting times including distance**

|  | (1)  Waiting Time | (2)  Waiting Time |
| --- | --- | --- |
| **Income deprivation (baseline 1 - least deprived)** |  |  |
| 2nd income deprived quintile | 0.136 | -0.0100 |
| 3rd income deprived quintile | 0.236* | 0.193 |
| 4th income deprived quintile | 0.426** | 0.0614 |
| Most income deprived quintile | 0.648*** | 0.243 |
| **Distance (baseline <= 5km)** |  |  |
| ]5,11] | 0.0695 | 0.183 |
| ]11,21] | -0.101 | 0.704 |
| ]21,50] | -0.119 | 0.807 |
| Above 50 | -0.113 | -2.792* |
| Hospital fixed effects | Yes | No |
| HRGs effects | Yes | Yes |
| Observations | 72,816 | 72,816 |
| Adjusted *R*^2^ | 0.164 | 0.057 |

Notes: * *p* < 0.05, ** *p* < 0.01, *** *p* < 0.001. Linear regression model with clustered robust standard errors at the hospital level. All models include financial year and month fixed effects. COVID-19 period: February 2020 to March 2022. The full model specification is presented in (1), i.e., it all covariates (age, ethnicity, secondary diagnosis, past emergency readmissions, HRGs and hospital fixed effects). Model (2) includes all covariates except the hospital fixed effects. Distance bands were created based on the percentiles 25, 50, 75, and 95 of the distance distribution. HRGs: Healthcare Resource Groups. “Distance” is the distance between the patient location and hospital location.
